# Impact Analysis of Flow Shaping in Ethernet-AVB/TSN and AFDX from Network Calculus and Simulation Perspective

**DOI:** 10.3390/s17051181

**Published:** 2017-05-22

**Authors:** Feng He, Lin Zhao, Ershuai Li

**Affiliations:** School of Electronic and Information Engineering, Beihang University, Beijing 100191, China; zhaolin@buaa.edu.cn (L.Z.); Ershuai_li@hotmail.com (E.L.)

**Keywords:** real-time Ethernet, network performance evaluation, credit based shaper, AVB, AFDX, TSN, end-to-end delay, network calculus

## Abstract

Ethernet-AVB/TSN (Audio Video Bridging/Time-Sensitive Networking) and AFDX (Avionics Full DupleX switched Ethernet) are switched Ethernet technologies, which are both candidates for real-time communication in the context of transportation systems. AFDX implements a fixed priority scheduling strategy with two priority levels. Ethernet-AVB/TSN supports a similar fixed priority scheduling with an additional Credit-Based Shaper (CBS) mechanism. Besides, TSN can support time-triggered scheduling strategy. One direct effect of CBS mechanism is to increase the delay of its flows while decreasing the delay of other priority ones. The former effect can be seen as the shaping restriction and the latter effect can be seen as the shaping benefit from CBS. The goal of this paper is to investigate the impact of CBS on different priority flows, especially on the intermediate priority ones, as well as the effect of CBS bandwidth allocation. It is based on a performance comparison of AVB/TSN and AFDX by simulation in an automotive case study. Furthermore, the shaping benefit is modeled based on integral operation from network calculus perspective. Combing with the analysis of shaping restriction and shaping benefit, some configuration suggestions on the setting of CBS bandwidth are given. Results show that the effect of CBS depends on flow loads and CBS configurations. A larger load of high priority flows in AVB tends to a better performance for the intermediate priority flows when compared with AFDX. Shaping benefit can be explained and calculated according to the changing from the permitted maximum burst.

## 1. Introduction

Classical vehicle networks can only provide limited bandwidth for control signal transmission through serial bus, such as CAN (Controller Area Network) and FlexRay. With in-car multimedia systems fast development and in-car navigation and intelligent driver assistance system appearance, the bandwidth requirements for vehicle electronic systems are increasing drastically. The biggest difference apart from the classical networks is there will be multiple traffic types, including control signals, audio signals and video signals generated or needed by distributed cameras, sensors and infotainment devices [1]. Limited by the bandwidth and multiple access conflicts, bus systems cannot cope with such great bandwidth requirements. Thus, new networking solutions are needed for future vehicle networks. The most promising solutions for vehicle network upgrading are based on switched Ethernet. Three main technologies are considered, i.e., AFDX (Avionics Full DupleX switched Ethernet), TTEternet (Time-Triggered Ethernet) and Ethernet-AVB (Audio Video Bridging), as well as its further development TSN (Time-Sensitive Networking). An important difference between these technologies concerns the scheduling of flows. AFDX [2] implements a non-preemptive mixed FP/FCFS (Fixed Priority/First Come First Served) scheduling in each output port. Ethernet-AVB [3,4,5,6] implements Credit-Based Shapers (CBS) on top of FP/FCFS. In contrast to these solutions, TTEthernet [7] adopts time-triggered scheduling to achieve strict timing transmission based on a mixture infrastructure to support TT, Rate-Constraint (RC) and Best-Effort (BE) traffics.

Much research work concerns performance comparisons among the three networking solutions, as well as IEEE 802.1Q, with different evaluating approaches, such as simulation method and analytic method. Ethernet-AVB, as well as its further proposal TSN, shows great interests and attractions to satisfy the increasing and variable transmission requirements for vehicle networks. The major challenge for the adoption of Ethernet-AVB/TSN in the industrial control domain lies in the impact analysis of its flow shaping mechanism and further the effects of its bandwidth allocation, in particular because the resources in industrial systems are limited, which lead us to the comparison research.

### 1.1. Related Work

AFDX [2] is a switched Ethernet network and designed to provide deterministic transmission guarantees especially for avionics context. It has the same physical layer as the standard Ethernet and adopts static definition for flows. Every flow should be restricted within a bandwidth envelope defined by a corresponding Virtual Link (VL) from its source and VLs can be seen as the logical bandwidth providers for flows. When flows aggregate into switches, they will be forwarded to destinations statically. AFDX implements a non-preemptive mixed FP/FCFS scheduling in each output port. A priority is assigned to each VL, as well as to each flow. At each output port the scheduler always transmits the pending frame with the highest priority. For a given priority level, frames are treated in a first come first served manner. Existing AFDX switches only implement two priority levels. New switches are envisioned with an increased number of levels, even to support the traditional Best Effort flows.

Ethernet-AVB [3,4,5,6] is a set of IEEE standards and designed to provide quality of service mechanisms for low latency communication especially for time sensitive flows with controllable latencies. Like AFDX, Ethernet-AVB adopts the same physical layer definition, which makes it easy for industrialization. However, Ethernet-AVB implements different scheduling strategies to make it more available for audio and video transmission. In AFDX, the output port scheduling can lead to high delays for low priority flows if large flow bursts from high priority ones take place usually. Ethernet-AVB implements Credit-Based Shapers (CBS) on top of FP/FCFS, which can partly mitigate this problem. A credit is associated to each of the two highest priority levels. A send slope and an idle slope are associated to each credit. The pending frame with highest priority can be transmitted only if there is remaining credit for the corresponding priority level. This mechanism prevents bursts of flows with high priorities. The new proposals for AVB develop into TSN [8,9,10]. A key new feature of TSN is the definition of new traffic shaping mechanisms [11] to accommodate strict real-time transmission with deterministic end-to-end delays.

As a real-time extension to standard Ethernet, TTEthernet [7] considers three kinds of flows: rate constrained flows, which are comparable to AFDX virtual links with a similar flow shaping mechanism; best effort flows with the lowest priority; and time triggered (TT) flows, which are assigned with dedicated slots at each output port along their path. Thus, TT flows have the highest transmission guarantees because the pre-designed slots can avoid flow conflicts throughout the whole networks. The next generation of AVB also considers this kind of scheduling method to meet the strict timing requirements for time-critical traffics, especially in its new traffic shaping mechanism: Time-Aware Shaper (TAS) [9]. 

Even though Ethernet-AVB/TSN, AFDX and TTEthernet adopt the same physical layer standard, they implement different scheduling methods to achieve transmission guarantees, which will lead to different networking performances. It is meaningful to make their performance comparisons and investigate these differences. 

The evaluation of Ethernet-AVB/TSN mechanisms has been addressed in the scientific literature. Some papers propose comparisons between Ethernet-AVB/TSN and TTEthernet [12,13,14,15]. In particular, Alderisi et al. [15] proposes a ST (Scheduled Traffic) scheduling strategy for Ethernet-AVB, which also is a kind of time-triggered communication, and Meyer et al. [14] studies the coexistence condition of synchronous and asynchronous flows in Ethernet-AVB where synchronous flows are transmitted according to pre-designed sending windows. It shows the time-triggered strategy greatly enhances the transmission guarantee ability for strict timing flows. IEEE 802.1Qbv [9] implements its philosophy by time-aware transmission gates. However, different arrangements of time-triggered windows will bring different influence to other priority flows [14], as well as the guard band phenomenon. IEEE 802.1Qbu [10] might partly mitigate the problem by preempting a frame in transmission. Still, there exist some other considerations to reduce the guard band size [16]. Other papers compare Ethernet-AVB with the standard Ethernet technology. In [17], it is shown that Ethernet-AVB does not reduce the worst-case delay for highest priority flows. In [18], authors explain that end-to-end delays and packet loss ratios of Ethernet-AVB flows are independent of network load, which is not the case with IEEE802.Q. To some extent, the results in [18] show the transmission stableness of Ethernet-AVB is owing to its logical bandwidth allocation method. Finally, some authors consider the integration of Ethernet-AVB in the context of avionics [19,20,21], as well as the comparisons [22] between Ethernet-AVB and AFDX since the latter has been successfully applied to avionics contexts for years. In particular, Geyer et al. [19] compares several common scheduling strategies with Ethernet-AVB and suggests the scheduling architecture proposed in IEEE 802.1Qav [6] could be a possible candidate for an evolution of AFDX when mixing avionic flows with other types of flows. In [20], authors establish a basic framework in order to use Ethernet-AVB in avionics, including a flow mapping strategy and suggest that Ethernet-AVB CBS algorithm and clock synchronization mechanism can be implemented according to aeronautic requirements. In [22], four Ethernet-based protocols, including AFDX, TTEthernet, EtherCAT (Ethernet for Control Automation Technology) and Ethernet-AVB, are compared from cost, physical layer, topology, redundancy, security, etc. However, still it is lack of further discussion on different priority flows and suggestions on how to configure CBS parameters.

For the worst-case delay analysis of Ethernet-AVB/TSN, the major challenge is to acquire a tight enough upper bound. Existing methods can be divided into four kinds: Compositional Performance Analysis (CPA), Network Calculus (NC), Trajectory Approach (TA) and miscellaneous methods. The first set of papers concern CPA method [23,24,25,26,27]. CPA is based on an iterative approach to seek the maximal stable delays in a busy period according to worst-case arrival sequences of all interfering flows. To some extent, CPA can be seen as a variant of holistic method, which has been applied into AFDX successfully [28]. In particular, Thiele et al. [26] propose an analytical model for Burst-Limiting Shaper (BLS) in TSN considering all blocking effects, especially those of same-priority flows. The second set of papers relates to network calculus method. Queck et al. [29] expresses a worst-case delay analysis model according to the basic network calculus, in which the arrival curve and minimal service curve are defined for each Stream Reservation (SR) flow, also it demonstrates an industrial case for Ethernet-AVB applications. In [30], authors make a further discussion on Ethernet-AVB network calculus models. Besides the minimal service curve, another two service curves are defined, including the strict minimal service curve and the maximal min-plus service curve. Combining the three service curves can get a much tighten end-to-end delay analysis. The third set of papers consider using trajectory approach to tighten the upper bound of flows in Ethernet-AVB with serialization constraints as shown in [31]. Finally, there still exist some miscellaneous analytical methods. In [17], a timing analysis of Ethernet-AVB is presented both with simulation and analytical results. The computing of queuing delays mainly refers to the standard analysis method mentioned in IEEE 802.1Qav [6]. In [32], authors propose a method by defining an eligible interval to tight the upper bound of worst-case delays. 

Besides these evaluation studies for Ethernet-AVB/TSN, there are few papers considering bandwidth allocation method. In [33], authors discuss an optimal bandwidth allocation method for scheduled flows by adjusting their Maximum Transmit Unit (MTU) size for TSN. Results in [33] show the best ratio for scheduled flows is around 7%, but still it needs more experiments for the detailed parameters setting.

### 1.2. Motivations and Contributions

This paper attempts to investigate the impact by CBS and its bandwidth allocation strategy. Our study is motivated mainly by three facts. 

Firstly, the impact of CBS to the intermediate priority flows is still unclear, especially in contrast to AFDX. Ethernet-AVB/TSN includes Stream Reservation (SR) as well as best-effort flows (BE). SR flows can be allocated to SR-A class (higher priority) and SR-B class (lower priority). At any time, the oldest pending frame from SR-A gets ready to be transmitted, provided that there is remaining credit for SR-A (CBS mechanism). A similar rule is applied to SR-B. Since SR-A class flows cannot fully use all bandwidths and the sum of SR-A and SR-B idle slopes is recommended not to exceed 75% of the total bandwidth, it is expected that lower priority flows (best effort) benefits from this mechanism, while higher priority flows (SR-A) experiment longer delays. The effect of CBS on SR-B flows is still not so obvious. It should depend on the slopes defining credits for SR-A and SR-B flows as well as the effective bandwidth of SR-A flows. This kind of analysis work has not been found in the literatures.

Secondly, the shaping benefit from CBS to other priority flows has not been analyzed yet. Indeed, a tight idle slope for a certain SR class will bring strict flow control for itself as well as leave more transmission opportunities for other priority flows. It can be seen as the shaping benefit from CBS mechanism. One criterion for the benefits can be the difference among the end-to-end delays of flows and simulation methods can be used to get the detailed data. However, we try to explain the benefits from network calculus aspect, especially from the changing of the maximum flow bursts.

Thirdly, there is still a lack of detailed bandwidth allocation strategies for CBS parameter configurations. Although the specifications defined in Ethernet-AVB/TSN can guide the design of its networking, they do not provide detailed utilization suggestions, such as the bandwidth allocation methods, especially when the networking resource is not adequate enough. In this paper, we try to give some suggestions to CBS idle slope setting mainly from two parts: shaping restriction and shaping benefit. The former dominates flow delays and the latter could bring some benefits, and the finally delays can be seen as a balance of these two aspects. 

In this paper, we focus on the analysis for the impact of the credit based shaper. The contribution of this paper firstly is to evaluate the impact of CBS on SR-B flows in a typical automotive case study by using a simulation approach, and also figure out the performance crossing points between Ethernet-AVB and AFDX. The second main contribution of our work is to interpret the benefits of CBS from network calculus aspect, which might be the first work to combine the worst-case analytic method with the simulation approach. As a third contribution, we give the configuration suggestion on the setting of CBS bandwidth allocation, according to the analysis results from CBS impact. 

In the rest of this paper, we recall main characteristics of AFDX and Ethernet-AVB, as well as its further development TSN. Then, we present performance results of the two networks according to an industrial case study. Further results obtained when modifying flows parameters (load of highest priority frames) are then discussed. After that, we will focus on the impact analysis of CBS mainly from shaping restriction and shaping benefit the two parts, and an analytical method according to integral operation is proposed to calculate the detailed shaping benefits. Finally, CBS bandwidth allocation suggestions are discussed.

## 2. Background

### 2.1. AFDX Network

AFDX and Ethernet-AVB are both switched Ethernet technologies. AFDX [2] has become the de facto standard in the context of avionics communications. It takes into account avionics constraints. Thus, end-to-end traffic characterization is made by the definition of Virtual Links (VL). As standardized by ARINC 664 (Aeronautical Radio Inc.: Annapolis, MD, USA), VL is a concept of unidirectional virtual communication channel, including only one source end system and one or more destination end systems (multicast mode). At the source end system, flows are restricted and shaped by VL logical bandwidth (pre-allocated bandwidth by system designer). At the switch output port, flows are forwarded by VL traffic policing strategy and fixed routing tables. 

**Figure 1:**
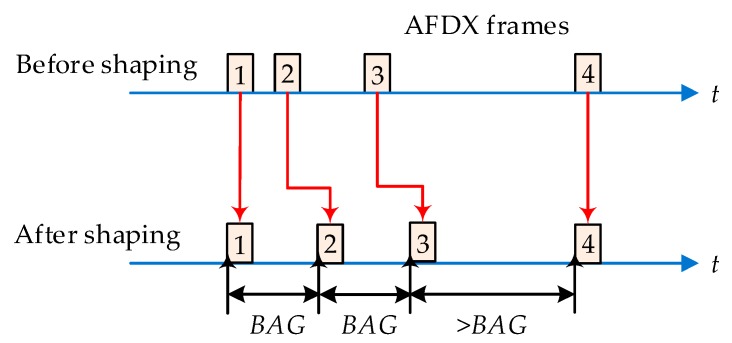
*BAG* shaping process in AFDX.

A VL is typically characterized by two parameters: Bandwidth Allocation Gap (*BAG*) and maximum frame length *S_max_*. *BAG* defines the minimum duration between two consecutive frames of the corresponding VL, and *S_max_* restricts the maximum permitted frame length. According to ARINC 664 specification, *BAG* is a power of 2 comprised between 1 ms and 128 ms. During some extended applications of AFDX, the setting of *BAG* can be any multiple of 0.5 ms within the scope from 0.5 ms to 128 ms. If the actual frame length is larger than *S_max_*, it should be divided into several sub-packets. According to these parameters, we can obtain the maximal logical bandwidth for a given VL as *bandwidth* = *S_max_/BAG*. This kind of flow shaping method determines the guarantee ability of AFDX end-to-end performance, which is shown in Figure 1. Besides, AFDX adopts priority-scheduling strategy (High Priority: HP and Low Priority: LP) in each output port. The coming frames with the same priority obey FCFS scheduling. An extended AFDX including more than two priority levels is envisioned for future aircrafts. In this paper, we consider three priority levels in our experiments to let AFDX support BE flows. There are many precise analysis methods for the upper bound estimation of the end-to-end delays in AFDX, such as Network Calculus [34,35], Trajectory Approach [36,37] and Holistic Method [28].

### 2.2. Ethernet-AVB Network

**Figure 2:**
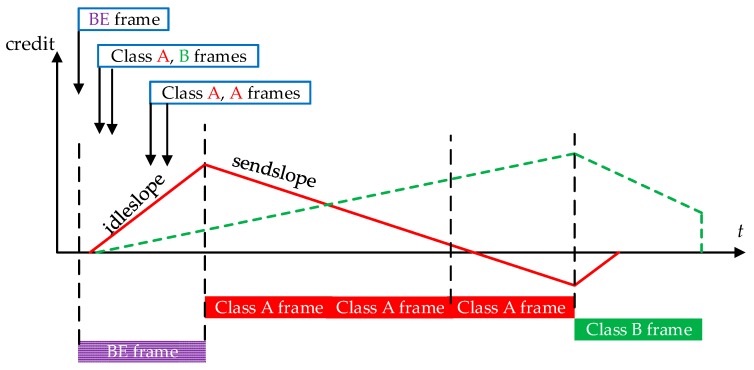
Credit-based shaper process in Ethernet-AVB.

IEEE 802.1 Audio Video Bridging (Ethernet-AVB) is a suite of specifications for low latency flows over IEEE 802 networks. IEEE 802.1AS [4] defines the synchronization method over distributed end systems with a steady-state clock synchronization accuracy of 1 µs or better over seven hops. IEEE 802.1Qat [5] defines a stream reservation protocol with a detailed stream registration and bandwidth pre-allocated method among stream paths over switches (AVB bridges) between sender and receiver (s). IEEE 802.1BA [3] defines the overall Ethernet-AVB systems and default configurations. In this paper, we are mainly interested in IEEE 802.1Qav [6], which specifies queuing and forwarding rules for Ethernet-AVB switches. As previously mentioned, Ethernet-AVB implements a priority queuing scheduling. A credit-based shaper (CBS) is associated with each of the two highest priority levels, namely Stream Reservation classes A and B (SR-A and SR-B). CBS process is illustrated in Figure 2. Flows not belonging to SR classes are treated as BE class with the lowest priority and can only be transmitted out when there are no SR class frames waiting in the queuing buffers or SR class credits are not enough for transmission.

Focused on SR class flows, the transmission is only allowed when:

1. the corresponding credit is positive or equal to zero;
2. there is no higher priority class frame in its queue or the corresponding higher priority class credit is not enough for transmission (higher priority CBS blocks its own frame transmissions due to insufficient credit);
3. no other frame (including lower priority frame) is currently being sent (no preemption).

The corresponding credit allocated to a given class starts at 0. It decreases at the rate of *sendslope* when a frame from this class is being transmitted. After the transmission, it increases at the rate of *idleslope*. If the credit has grown to zero and there are no frames waiting in the corresponding queuing buffer, then the credit will be set to zero and wait for frames coming. If the transmission of SR class frame is blocked, its credit keeps growing at the rate of *idleslope* and can exceed 0. Under this mechanism, SR class frame is scheduled according to priority strategy with CBS algorithm. The usage of *sendslope* and *idleslope* provides great flexibility for Ethernet-AVB flow control. *Sendslope* and *idleslope* are deduced from the bandwidth fraction allocated to the class, and should obey *sendslope* = *idleslope* − *linkspeed*. Usually, the physical link speed is 100 Mbps. If we want to accelerate the transmission speed for some kind of SR class flows, we can increase the corresponding *idleslope* to ensure its credit to recover quickly. On the other hand, if we want to slow down the transmission speed, we can decrease its corresponding *idleslope*. 

According to 802.1Qat [5], it uses CMI (Class Measurement Interval), MFS (Maximum Frame Size) and MIF (Maximum Interval Frame) to define Ethernet-AVB flows. For a given flow, there are at most MIF frames during one CMI, and MFS defines the maximum frame length. In other words, CMI can be seen as a periodical time interval in which talker (the source) can generate up to MIF frames at most with frame length no longer than MFS. Since there is no further restriction for the offsets setting among the different MIF frames, the transmission of MIF frames could be non-periodic. For SR-A class flows, the standard CMI is 125 µs; for SR-B class, the standard CMI is 250 µs. In order to expand the application scope, in some detailed configurations, CMI can be assigned with different values, such as application period. According to 802.1Qat, the long term bandwidth requirement of one Ethernet-AVB flow is: *bandwidth* = (*MIF* × *MFS*)/*CMI*. 

### 2.3. Time-Aware Scheduling and Shaping

Due to unpredictable arriving instants of asynchronous flows, there will always be unexpected interference among different flows, which leads to great uncertain for flows forwarding, especially when considering the worst-case scheduling scenario. Since Ethernet-AVB supports distributed synchronism over Ethernet networks, some extension solutions with fully deterministic transmission for strictly real-time applications have been proposed. These solutions are gradually concentrated into the second generation of AVB, further named as TSN (Time-Sensitive Networking) [8].

TSN introduces a new Control-Data Traffic (CDT) [11] class, assigned as the highest priority (even higher than SR-A and SR-B), to satisfy hard real-time transmission requirements. In order to improve CDT class transmission latency and jitter, new shaping mechanisms have been proposed. One of the these new shapers is Time-Aware Shaper (TAS) and it adopts a gate operation to ensure that only one traffic class (or a set of traffic classes) has access to the network at some specific time windows [8]. In other words, critical flows belonging to this specified class are scheduled according to time-triggered mechanism, which is quite similar to TTEthernet scheduling strategy.

According to TAS mechanism, the most critical flows can be assigned with CDT priority, whose transmission windows are pre-designed dedicatedly to avoid flow conflicts throughout the whole networks by synthesizing of time-aware transmission gates [38]. These gates will be open at the scheduled time and closed otherwise by a gate driver according to a configured schedule named as Gate-Control Lists (GCL). Thus, TAS defines a fixed and periodic scheduling schema for CDT flows. The list of gate control is mainly composed of two fields: a time indicating the next gates operating, and a binary gate configuration representing an open or close behavior for the corresponding traffic class. CDT class frames can only be sent out during CDT gates opening windows.

For SR frame queuing behaviors, there are two integration modes: non-preemption and preemption [10]. Non-preemption could introduce a guard band to avoid interferences from other priority flows to CDT flows. In the worst-case scheduling scenario, the guard band is equal to the longest frame length among interfering flows, which finally might result in great delay to AVB and BE class flows. For preemption mode, the transmission of a low priority frame can be interrupted by CDT frames and further be recovered just after these CDT frames.

**Figure 3:**
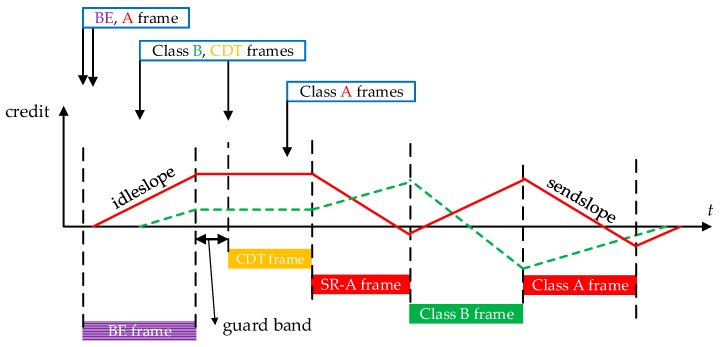
Credit-based shaper process in TAS with non-preemption mode.

With TAS mechanism, the original scheduling strategy and CBS shaper function in AVB should make some modifications. Scheduler first checks time-aware transmission gates during the whole scheduling process. In CDT gates opening windows, only the corresponding CDT class frames can be sent out. Credits associated to AVB SR are frozen during these windows as the corresponding SR gates are closed. In SR gates opening windows, SR frames can be scheduled out according to AVB CBS algorithm. One example of the CBS rule in TAS is illustrated in Figure 3. We can also find the guard band in Figure 3 since it shows the non-preemption mode for SR frame queuing behaviors. If the preemption mode is considered, the credit consuming and recovering process are different from Figure 3. 

## 3. Industrial Case 

**Figure 4:**
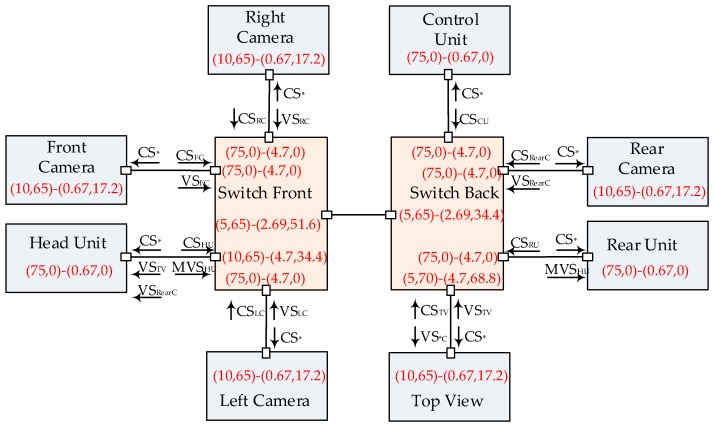
Ethernet-AVB industrial case with Control Signal (CS), Video Signal (VS) and Multimedia Video Signal (MVS) flows and *idleslope* and throughput at each port(*idleslope* SR-A, *idleslope* SR-B)–(throughput SR-A, throughput SR-B).

An industrial case study has been chosen for Ethernet-AVB/TSN performance evaluation. It is based on an automotive Ethernet-AVB network (presented in [29]), which is shown in Figure 4.

Eight end systems are connected to two Ethernet-AVB switches: Switch Front (SF) is in charge of flow forwarding within the front part and Switch Back (SB) is for the back part. Four automotive cameras are integrated for driver assistance: one is in front of the car, one is in the back, and the other two are installed at each side (Left Camera: LC and Right Camera: RC). These cameras gather Video Signals (VS) and then send them to Top View (TV). TV end system merges all these four video signals and then sends resulting video signal to Head Unit (HU). HU end system displays these results simultaneously with Rear Camera (RearC) video signal. Besides, HU emits additional Multimedia Video Signal (MVS), such as navigation information, to Rear Unit (RU). Control Unit (CU) is in charge of the whole network management and all Control Signals (CS) are broadcasted to every end system. *Idleslope* parameters for SR-A and SR-B and the actually total load per class are also shown in Figure 4. For example, for Right Camera, the *idleslope* of SR-A class is 10 Mbps and the *idleslope* of SR-B class is 65 Mbps. The actual total load of SR-A is 0.67 Mbps and the total load of SR-B is 17.2 Mbps. All of these *idleslope* settings were taken from [29].

The detailed flow parameters are shown in Table 1, including CMI and MIF. For instance, Video Signal generates at most 46 frames of 1522 bytes every 33 ms. Here, CMI as 33 ms is an extension of the standard definition in Ethernet-AVB. All of these flows are mapped into three kinds of classes: SR-A, SR-B and BE. Control signals are strictly time-sensitive and mapped into SR-A class; video signals are forwarded with intermediate priority as SR-B class; multimedia signals can tolerate relatively larger transmission uncertainty and are assigned as BE flows. The basic link speed is 100 Mbps for all physical links. According to the configuration, the most loaded link is SB→TV (73.5%) from Switch Back to Top View and the average load is about 20.6% (including both directions). According to the configuration, the bandwidth reservation for both SR-A and SR-B classes in the source end systems are quite adequate for CS and VS flows transmissions, since one CS flow only occupies 0.67 Mbps and one VS flow occupies 17.2 Mbps bandwidth. The bottleneck of the networking case lies in the following ports: port from SF to HU, port from SF to SB, port from SB to SF and port from SB to TV. For example, the bandwidth reservations for SR-A and SR-B at port SB→TV are 5 Mbps and 70 Mbps respectively in contrast to the actually strict bandwidth requirements as 4.70 Mbps and 68.8 Mbps. Thus, the reservation margin degrees are only 5.9% and 1.7% at this port. In Section 4.3 and Section 5.1, the original *idleslope* settings at these bottleneck points will be changed to meet larger reservation margins or big frame length experiment requirements.

Our goal is to compare this Ethernet-AVB architecture with an equivalent AFDX one, as well as its time-aware scheduling and shaping mechanism. For the comparison between the standard Ethernet-AVB and AFDX, Ethernet-AVB switches are replaced by AFDX switches implementing three priority levels to support the best effort flows. Control signal are assigned with the highest priority (HP for VLs) while Video signals are assigned with the medium priority (LP for VLs) and MVideo signals the lowest one (BE). For the comparison between the standard Ethernet-AVB and its further proposal TAS, Ethernet-AVB switches and end systems will perform time-triggered scheduling strategy to simulate the behaviors of time-aware transmission gates. Control signal are assigned with CDT priority while Video signals and MVideo signals hold their original ones.

According to Ethernet-AVB protocol, there will be multi-frames in each CMI. For example, each Control Signal flow can accommodate 10 frames in every 10 ms. However, one VL in AFDX cannot accept so many sub-flows simultaneously because of the restriction by *BAG*. Besides, a single VL with a *BAG* of CMI/MIF would be less bursty. In this paper, MIF VLs with a *BAG* equal to CMI are defined for each type of flow. For example, 10 VLs with a *BAG* of 10 ms and a maximum frame length of 64 bytes are defined for one Control Signal flow. From the overall perspective, this mapping method can get the same utilizations and similar flow interference behaviors.

Both Ethernet-AVB (as well as TAS) and AFDX architectures are simulated using a self-designed tool based on OMNet++ V4.5. It was modeled base on the INET-Framework, which provides the implementation for the physical and MAC layer. The upper traffic shaping and scheduling layer were developed according to the basic philosophy of different networking protocols. Technological latency of Ethernet-AVB/TSN as well as AFDX switches is 8 µs. Routing of flows obeys the shorted-path algorithm, and duplication of packets for multicast occurs at each fork along flow path to reduce bandwidth waste.

For the comparison between the standard Ethernet-AVB and AFDX, we assume that the different flows are not synchronized. This is obviously the case with AFDX (no global clock). However, no information is provided in [29] about synchronization. Secondly, the operation of the standard CBS mechanism does not need the global time synchronization throughout the whole network. Thirdly, the random arrival of MIF frames during one CMI looks more like asynchronous behaviors. In order to make a fair comparison, this desynchronization of flows is modeled by random offsets associated to the different flows. All time-sensitive flows are periodically generated with a jitter of up to 1 ms. Best effort flows are exponentially generated. The detailed flow generating method can be found in Appendix A. 

For the comparison between the standard Ethernet-AVB and its further proposal TAS, we assume that the Gate-Control Lists (GCL) in TAS are given and the sending and forwarding behaviors of all CDT priority flows (CS flows) are synchronized. To minimize the influence from CDT priority flows to other priority flows, all CDT frame sending windows are allocated one by one as much as possible. In the actual simulation setups, the gap between two adjacent CDT frames sending points is 10 µs compared to 6.72 µs of the actual transmission requirement. For the other priority flows, such as SR-B and BE, the simulating methods are the same with those in the standard Ethernet-AVB.

## 4. Performance Comparison

### 4.1. A Simple Case Illustration

**Figure 5:**
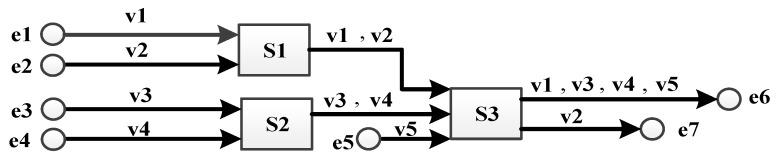
A simple AFDX case including five VLs and three switches.

First, a simple networking case [37] is studied to verify our simulation model. Five VLs (v1 to v5) are forwarded in an AFDX network constituted by seven end systems (e1 to e7) and three switches (S1 to S3) and the basic link speed is 100 Mbps. All VLs are configured with the same parameters: *S_max_* = 500 bytes and *BAG* = 40 ms. Thus, the basic wire transmission time for any frame with the permitted maximal packet length is 500 bytes × 8/100 Mbps = 40 µs in any output port without considering frame interfering. According to [37], technological latency of all switches is 16 µs. The networking topology is shown in Figure 5.

The statistical average delay and observed maximal delay from simulation, as well as the real worst-case delay mentioned in [37], are shown in Table 2.

The maximal delay of a flow is collected by observing the maximum value among all delays. As the nature of simulation method, it might not be the real worst-case delay since simulation cannot assure all flows just happen to experience their worst-case blocking scenario. According to simulation results for the simple networking case, the observed maximal delays are close to the real worst-case delays. Besides, the average delays are also close to the sum of the strictly basic transmission time at output ports plus the assigned technological latency. It can be explained by the low bandwidth occupation since the most loaded link (from S3 to e6) only has 0.4% bandwidth usage (400 Kbps bandwidth requirement versus 100 Mbps link speed). 

### 4.2. General Simulation Results

As mentioned above, MIF VLs are adopted to simulate one Ethernet-AVB/TSN flow with MIF frames in one CMI. During the statistic process, we use the average delay of MIF VLs in AFDX to represent the final average delay of one flow, and the maximum delay among all MIF VLs to stand for the final maximal delay. 

**Figure 6:**
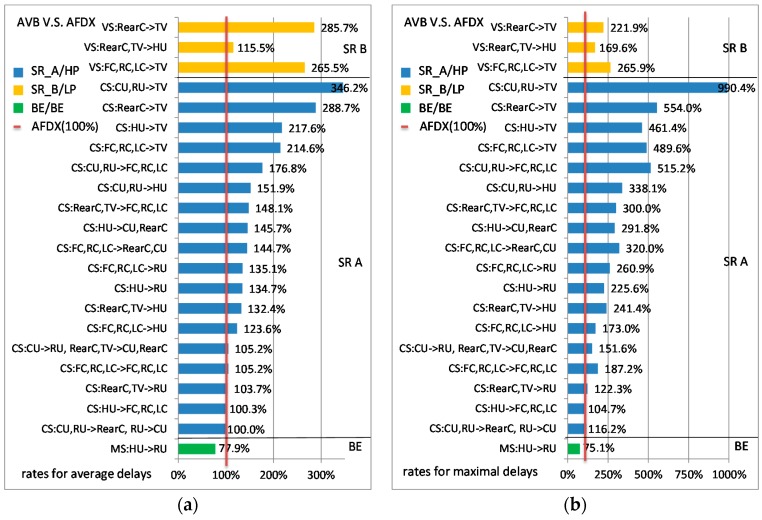
Delay ratios (average delay ratios and maximal delay ratios) for the standard AVB over AFDX: (**a**) average delay ratios of the standard AVB compared to AFDX; and (**b**) maximal delay ratios of the standard AVB compared to AFDX.

The comparisons between the standard Ethernet-AVB and AFDX are shown in Figure 6. The simulations were executed 50 times for a duration close to three times the Least Common Multiple (LCM) of all flow periods and each time all the flows started with random initial offsets. Our experiments show the simulation results have a good stable state. The more execution times or longer simulation duration have little change to the final results. For example, the average delay after 60 executing times for a duration of five times the LCM only changes 0.1 µs or less. The influence to the maximal observed delay should be bigger, but our experiments show the changing is limited within 6 µs for all flows. AFDX delays are considered as the reference. Figure 6a,b gives for each flow the ratio of average AVB delay and maximal AVB delay when compared to AFDX ones. For a given flow, the ratio is obtained using the following equation.

(1) $$ratio\%=\frac{delay_{AVB}}{delay_{AFDX}}\times 100\%$$

For example, the ratios for VS:RearC,TV→HU are 115.5% in Figure 6a and 169.5% in Figure 6b. It means that, for this flow, the average AVB delay is 15.5% larger than the AFDX delay, but the maximal AVB delay is 69.5% larger than that. 

In this case study, AFDX gives smaller average delays and maximal delays than the standard Ethernet-AVB ones for both High priority (SR-A) and Low priority (SR-B) flows. The opposite situation is observed for BE flows. The overall trends of the ratios between average delay and maximal delay are similar. Besides, for a single flow, the ratio of its maximal delay compared to the corresponding average delay is shown in Table 3 for some example flows, as well as their detailed end-to-end delays.

Results for SR-A flows are not surprising. The extra Ethernet-AVB delay is due to the shaper. We can observe that when *idleslope_A_* is large, the difference between AFDX and AVB is small and it increases when *idleslope_A_* decreases. SR-A flows which only concern sub-parts (Front Part or Back Part) of the architecture experiment small difference between AFDX and AVB delays. These flows are transmitted using a large *idleslope_A_* (75 Mbps). Thus, the delay caused by SR-A shapers to these flows is small, especially when considered the average delays. For flows CS_FC_,CS_RC_,CS_LC_→HU, and CS_CU_,CS_RU_,CS_RearC_→TV, the *idleslope_A_* settings at the last output ports are relatively small (5 Mbps and 10 Mbps, respectively) and the latency caused by SR-A shapers constitutes the main part of the final end-to-end delays. For SR-A flows that cross the two sub-parts of the network, the small *idleslope_A_* setting (5 Mbps) for both directions between Switch Front and Switch Back is the main reason for delay difference.

Results for SR-B flows depend on the actual bandwidth of both SR-A and SR-B flows as well as shaper configuration. On the considered case study, AFDX gives smaller average delays and maximal delays. For flows VS_RearC_,VS_TV_→HU, Ethernet-AVB delays are much closer to AFDX delays than for flows VS_FC_,VS_RC_,VS_LC_→TV. The main reason for that is the bigger margin of *idleslope_B_* for the link from Switch Front to HU (65 Mbps setting, 34.4 Mbps strict requirement). For the link from Switch Back to TV, we have only 70 Mbps setting but 68.8 Mbps strict requirement.

Ethernet-AVB is better for BE flows. Indeed, Ethernet-AVB CBS could lead to a credit recovery time after each time-sensitive flow transmission as it consumes the credit. Credit recovery time is a time interval in which the associated credit recovers to zero according to its configured speed. If the credit is consumed to be less than zero, it needs a credit recovery time after the current transmission. During this time interval, no frames can be further scheduled out. This mechanism increases some transmission opportunities for BE flows. In the worst-case, the longest credit recovery time for SR credit starts from the end of a frame transmission with the longest frame length, and this frame just finishes its consumption of the credit from zero from the beginning. Since the consuming speed is *sendslope* and the recovery speed is *idleslope*, the longest recovery time is restricted by Equation (2) in which *T_transmission_* is the transmission time for the frame. After this recovery interval, the corresponding credit recovers to zero again.

*T_recovery_* = *T_transmission_* × (−*sendslope*/*idleslope*) (2)

**Figure 7:**
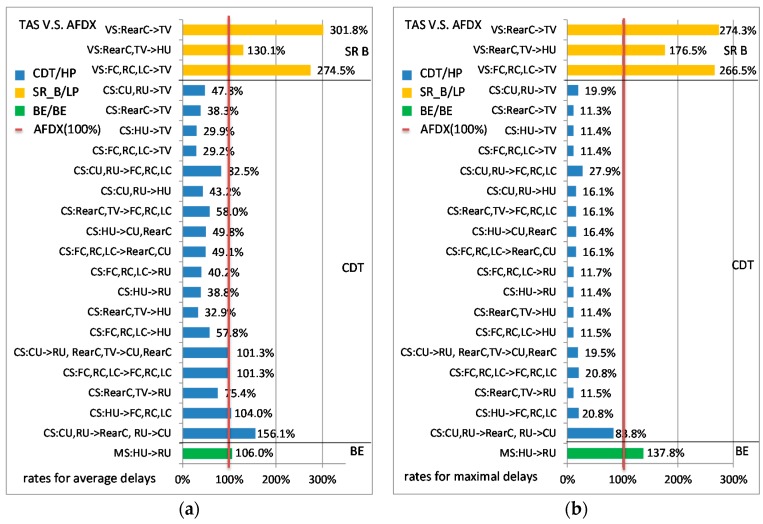
Delay ratios (average delay ratios and maximal delay ratios) for TAS over AFDX: (**a**) average delay ratios of TAS compared to AFDX; and (**b**) maximal delay ratios of TAS compared to AFDX.

The comparisons between the TAS and AFDX are shown in Figure 7. Similar to above, the simulations were executed 50 times. For SR-B flows, their initial offsets will be reset with random values each time. AFDX delays are still considered as the reference. Figure 7a,b gives, respectively, the ratios of the average delays and maximal delays for each flow in TAS compared to in AFDX. 

As the nature of time-triggered mechanism, all CS flows in TAS obtain perfect transmission stableness. The maximal delays of CS flows are quite close to their average delays. For example, the average delay of flow CS_FC_→TV is 45.8 µs and the maximal delay is 46.0 µs, which constitute the average delay ratio as 29.2% and maximal delay ratio as 11.4% in contrast to AFDX ones (shown in Figure 7). The difference between the average delay the maximal delay mainly lies in the precision of time synchronism. Besides, different arrangements of CDT scheduling windows will bring different end-to-end delays. This influence not only affects the delays of CDT priority flows, but also affects the delays of other priority flows. For SR-B flows, the average delay and maximal delay of every VS flow in TAS are larger than the corresponding delays in the standard Ethernet-AVB, as well as larger than those in AFDX. For example, the ratio for flow VS_Reac_→TV in the comparison of AVB versus AFDX is 115.5% (shown in Figure 6a) in contrast to 130.1% (shown in Figure 7a) in the comparison of TAS versus AFDX. In this case study, AFDX gives smaller average delay and maximal delay for BE flows than TAS does. Since the final delays of SR-B and BE flows seriously depend on the arrangements of CDT gates opening windows, we mainly focus on the comparison between the standard Ethernet-AVB and AFDX in the following sections.

### 4.3. Performance Crossing Point

**Figure 8:**
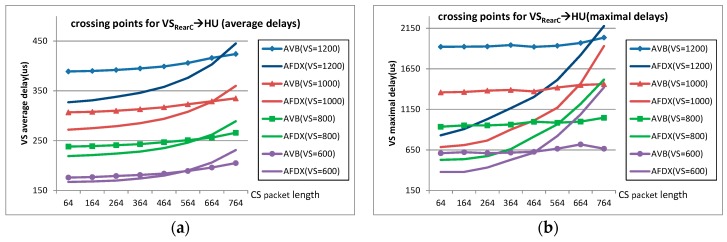

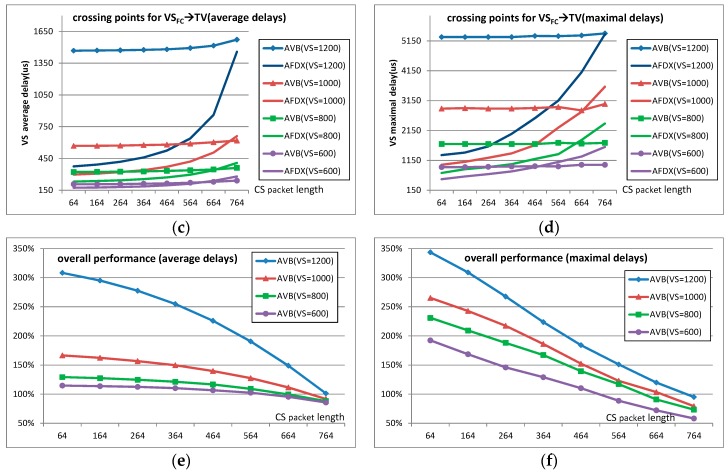
Performance difference for SR-B in Ethernet-AVB and LP in AFDX: (**a**) average delay crossing points for flow VS_RearC_→HU; (**b**) maximal delay crossing points for flow VS_RearC_→HU; (**c**) average delay crossing points for flow VS_FC_→TV; (**d**) maximal delay crossing points for flow VS_FC_→TV; (**e**) the overall average delay performance for SR_B/LP; and (**f**) the overall maximal delay performance for SR_B/LP.

In this section, further comparison is carried out to get the performance crossing point between the standard Ethernet-AVB and AFDX. Since Ethernet-AVB nearly always brings extra delays for SR-A in contrast to AFDX, the comparison is mainly focused on SR-B flow delays. Different *idleslope* settings for the most loaded ports SF→HU, SF→SB, SB→SF, and SB→TV are used to meet big frame length experiment requirements. These settings are (45 Mbps, 55 Mbps) for the four ports. For the sake of simplicity, BE flows are removed from the industrial case. We simulate considering CS flows with payload from 64 bytes to 764 bytes by step of 100 bytes and VS flows with payloads from 600 bytes to 1200 bytes by step of 200 bytes. For the most loaded case (CS flow lengths are all assigned as 764 bytes and VS flow lengths are assigned as 1200 bytes), the actually strict bandwidth requirements for CS flows and VS flows at port SB→TV are 42.78 Mbps and 53.53 Mbps which are also within the logical bandwidth envelops defined by the changed reservation setting (45 Mbps, 55 Mbps). Simulation results are shown in Figure 8. Each point in Figure 8 is achieved by simulating 50 times and each time all the flows started with random initial offsets.

Figure 8a,b, shows the average delay differences and maximal delay differences for flow VS_ReaC_→HU in AVB and in AFDX; Figure 8c,d shows these delay differences for flow VS_FC_→TV; and Figure 8e,f gives, respectively, the average delay ratios and maximal delay ratios for SR-B flows in Ethernet-AVB compared to LP flows in AFDX (there are 5 unicast VS flows and one multicast flow). We can observe that delays in both Ethernet-AVB and AFDX increase with a large CS frame length, but the incremental quantity in Ethernet-AVB is much smaller than that in AFDX. In other words, the transmission in Ethernet-AVB is more stable than in AFDX. For example, the delay variation of VS_ReaC_→HU with 1200 bytes in AVB is 9.0% (increasing from 389 µs to 424 µs, Figure 8a) when CS packet length changes from 64 bytes to 764 bytes. However, the corresponding delay variation of VS_ReaC_→HU in AFDX is 36.1% (increasing from 327 µs to 445 µs in Figure 8a). For flow VS_FC_→TV with 1200 bytes, the delay variation in AFDX is even large as 288.8% (increasing from 375 µs to 1458 µs in Figure 8c) in contrast to 7.2% (increasing from 1468 µs to 1573 µs in Figure 8c) in AVB with respect to the same load increasing of CS flows from 64 bytes to 764 bytes. This difference finally leads to performance crossing points between these two network solutions, not only for the average delay criteria, but also for the maximal delay criteria. For the case with small CS frame length, AFDX is better than Ethernet-AVB, as it was the case for the original study in Section 4.2. However, Ethernet-AVB becomes better for a large frame length. For example, when VS frame length is 1200 bytes, the performance crossing points of flow VS_RearC_→HU are around at 700 bytes (CS packet length) both for the average delay comparison and the maximal delay comparison. If VS is set with a smaller frame length, the performance crossing point will come earlier. For example, if VS frame length is 600 bytes, the crossing points are around at 550 bytes (CS packet length) for average delay and 460 bytes (CS packet length) for maximal delay.

The results in Figure 8 show that the Ethernet-AVB shaping for SR-A flows mitigates the impact of SR-A frame length on SR-B flows. Indeed, transmission of larger frames leads to longer recovery time for SR-A flows, and gives more opportunities to SR-B flows. Since no such mechanism exists in AFDX, medium priority flows are fully impacted by higher priority ones. Thus, for SR-B flows, the final delays depend on the balance between the transmission restriction by its own *idleslope* and the shaping benefit coming from SR-A CBS.

This kind of results are something like the comparison between Ethernet-AVB and standard Ethernet (with Priority Queuing strategy) shown in [17,18]. The difference lies in the exponential distributed method for flow generating in [17]. In fact, CBS shaping can result in big delays for high priority flows, but also provides some kind of guarantee ability for low priority flows. Unlike the standard Ethernet with PQ strategy, AFDX limits the entrance of flows at their sources by VLs and this is the reason why AFDX is called a deterministic networking solution, but still it is lack of a further method for flow controlling in the following switching nodes. Thus, the overall trends of the comparisons look similar, which reflects the fact that end-to-end delays of Ethernet-AVB flows are independent of network load, as shown in [18].

## 5. CBS Shaping Analysis

According to the discussion mentioned above, the final delay of a flow mainly depends on its own *idleslope* setting, also it will be affected by other priority flow’s shaping operation. The former can be seen as the shaping restriction by its own CBS, and the latter can be seen as the shaping benefit from other CBSs. In this section, we will make a further discussion on CBS shaping restriction and shaping benefit and give some analytic explanations about them. Average delay according to simulation will be used to check our fitting method, as it possesses much more stableness than the maximal delay from simulation.

### 5.1. CBS Shaping Simulation Results 

In order to obtain the detailed data of shaping restriction and shaping benefit to make further analytical study for them, a set of simulations have been done according to a series of *idleslope* setting experiments. 

**Figure 9:**
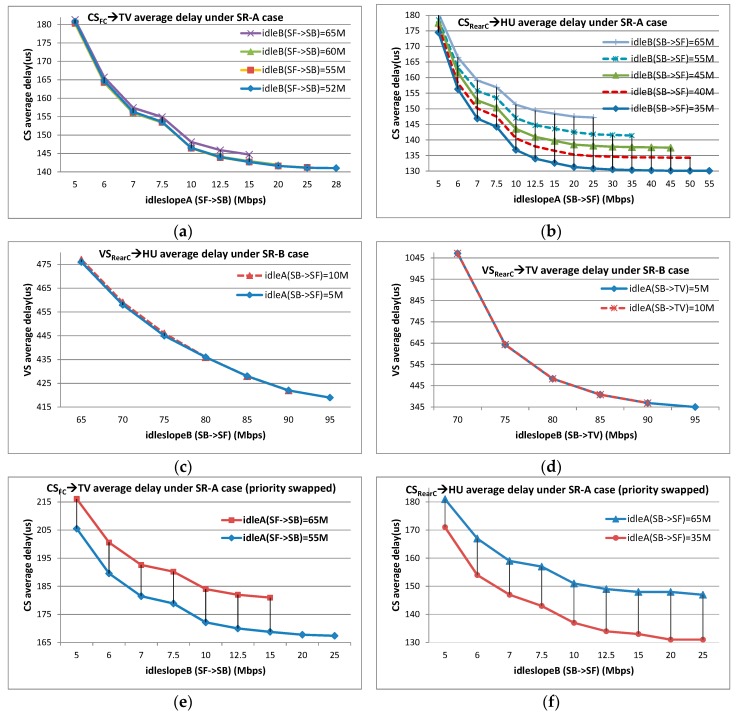
Impact simulation results: (**a**) CS_FC_→TV delay under SR-A evaluation case; (**b**) CS_RearC_→HU delay under SR-A evaluation case; (**c**) VS_RearC_→HU delay under SR-B evaluation case; (**d**) VS_RearC_→TV delay under SR-B evaluation case; (**e**) CS_FC_→TV delay under SR-A evaluation case (priority swapped); and (**f**) CS_RearC_→HU delay under SR-A evaluation case (priority swapped).

Two CBS scenarios are used for CBS shaping analysis according to the industrial case: one focuses on SR-A priority, and the other focuses on SR-B priority. For the case of SR-A evaluation, the shaping restriction by SR-A CBS is observed by increasing *idleslope_A_*, also the shaping benefit from SR-B CBS is investigated by decreasing *idleslope_B_*. The end-to-end delays of flows CS_FC_→TV and CS_RearC_→HU are observed based on a set of *idleslope* settings at ports of SF→SB and SB→SF. The initial *idleslope* settings at ports of SF→HU and SB→TV are changed into (35 Mbps, 65 Mbps) and (30 Mbps, 70 Mbps), respectively, to meet large bandwidth reservation requirements. The simulation results are shown in Figure 9a,b for flows CS_FC_→TV and CS_RearC_→HU, respectively, under SR-A evaluation case.

For the case of SR-B evaluation, the shaping restriction and benefit are also observed by increasing *idleslope_B_* and decreasing *idleslope_A_*. Flow VS_RearC_→HU and VS_RearC_→TV are checked under a set of *idleslope* settings at ports of SB→SF and SB→TV, and the initial *idleslope* at ports of SF→HU and RC→SB are changed into (5 Mbps, 95 Mbps) and (1 Mbps, 99 Mbps), respectively, for the same reason. The simulation results are shown in Figure 7c,d for flows VS_RearC_→HU and VS_RearC_→TV under SR-B evaluation case.

In addition, we have done some special experiments to investigate the detailed effect of shaping benefit by exchanging the priorities of all CS and VS flows. After this operation, all CS flows are configured as SR-B class and VS flows are configured as SR-A class, but the other configurations still hold the same as those under SR-A evaluation case. The simulation results are shown in Figure 9e,f for flows CS_FC_→TV and CS_RearC_→HU, respectively, under SR-A evaluation case; however, this time, the priorities of all CS and VS flows are swapped.

Each point in Figure 9 is obtained by simulating 50 times and each time all the flows started with random initial offsets.

Generally, SR CBS function dominates its flow delays. Both the average delays of flow CS_FC_→TV and CS_RearC_→HU decrease with big *idleslope_A_* settings, especially when *idleslope_A_* rises from a relatively small redundancy, such as from 5 Mbps to 7 Mbps (2.69 Mbps actual load for SR-A at ports SF→SB and SB→SF); but if the value of *idleslope_A_* is by far larger than the corresponding SR-A flows load, the gain by increasing *idleslope_A_* comes to be small. For example, when *idleslope_A_* at port SF→SB is larger than 15 Mbps, the average delay of CS_FC_→TV nearly keeps unchanged. The same trends are for flow VS_RearC_→HU and VS_RearC_→TV with different *idleslope_B_* settings (34.4 Mbps and 68.8 Mbps actual loads for SR-B at port SB→SF and SB→TV respectively).

In addition, SR flows can get benefits from the shaping by other priority CBS. With the same *idleslope_A_* setting, the average delays of flow CS_FC_→TV and CS_RearC_→HU can achieve better if *idleslop_B_* is set with a tighter value, especially when the bandwidth reservation for SR-A is large enough. However, this trend is not so obvious for SR-B flows. Even if *idleslope_B_* is set with a big value, such as 75 Mbps, the benefits from SR-A are no more than 2 µs. The reason may lie in two facts: on the one hand, the bandwidth reservation is still close to the strictly necessary requirement for VS flows, while, on the other hand, the load of SR-A flows is quite small. When the priorities of all Control Signal and Video Signal flows are swapped, it is easy to see the shaping benefits from high priority to low priority since the frame length and the load of SR-A flows are big enough and also there are adequate spaces to satisfy the large bandwidth reservation requirement for SR-B CBS. 

### 5.2. Shaping Restriction Analysis 

First, the shaping restriction by CBS will be studied since it dominates the end-to-end delay of flows. Delay variation rate will be observed according to the simulation results mentioned in Section 5.1, especially in Figure 9. It will be used to explain delay varying trend with respect to different *idleslope* setting. In the following section, a further suggestion about CBS parameters setting can be got from the investigation of delay variation rate. To achieve this, we use bandwidth reservation margin degree, instead of *idleslope* itself, to measure the tightness of CBS logical bandwidth, which can be defined as:

(3) $$deg=\frac{idleslope-band_{A/B}}{band_{A/B}}$$

where *band_A_* or *band_B_* represents the actually strict bandwidth requirement by SR-A flows or SR-B flows, respectively. For any given value of *idleslope* setting, such as 5 Mbps for SR-A CBS, the detailed bandwidth reservation margin can be calculated as *idleslope* − *band*_A/B_. Thus, margin degree can be got from the ratio of bandwidth reservation margin to the actually strict bandwidth requirement. In other words, it can be seen as a normalization for the extra reserved bandwidth. A smaller degree of reservation margin often means stricter flow control and severer transmission condition and vice versa.

Based on the concept of bandwidth reservation margin degree, the gradient of CBS shaping restriction can be further treated as the ratio of delay variation rate to bandwidth margin degree variation rate, which is shown as follows:

(4) $$\begin{array}{l} grad_{CBS}=\frac{\Delta delay\%}{\Delta deg\%}=\frac{(delay_{1}-delay_{2})/delay_{1}}{(deg_{1}-deg_{2})/deg_{2}}\%\\ \begin{array}{c} \;\;\;\;=\frac{(delay_{1}-delay_{2})/delay_{1}}{\left(idleslope_{1}-idleslope_{2})/(idleslope_{2}-band_{A/B}\right)}\% \end{array} \end{array}$$

Thus, the degree to decrease flow delay (measured as delay variation rate) by increasing the corresponding bandwidth reservation (measured as margin degree variation rate) is obtained according to Equation (4). Also Equation (4) can be seen as the gain from shaping restriction. Table 4 shows the calculated results for example flows. All delay data come from the simulation shown in Section 5.1.

Generally, the gain from shaping restriction deceases with large degrees of bandwidth reservation margin both for SR-A CBS and SR-B CBS. Though a larger bandwidth margin degree might bring more opportunity for flow transmission, the unit effectiveness of reducing delay decreases successively gradually when increasing a unit *idleslope* in successive. If the gain of 5% is considered as a threshold, the corresponding margin degrees for flows CS_FC_→TV and CS_RearC_→HU under SR-A evaluation case are around 2.7, no matter whether the priorities of flows are swapped. The corresponding values of *idleslope* are around 10 Mbps in contrast to 2.69 Mbps of the strict bandwidth requirements both at ports SF→SB and SB→SF. For SR-B flows, the maximal observed margin degrees are 1.62 and 0.31 for flows VS_RearC_→HU and VS_RearC_→TV at ports SB→SF and SB→TV, respectively. If the *idleslope* settings could be set with 90 Mbps or even higher, it can be predicted that the corresponding gains would still be remarkable. It is not surprise for the effect of shaping restriction since the margin degrees for SR-B CBS with these configurations are by far less than 2.7 as it has done for SR-A CBS. If the threshold is considered as 10%, the corresponding margin degrees for these observed flows are around at 1.7.

The gains of CBS shaping restriction in Table 4 also show the influence from different *idleslope* settings for other priority flows. A tighter *idleslope* setting for other priority flows could increase the corresponding gain from shaping restriction and vice versa. Since the margin degrees of 2.7 and 1.7 for thresholds 5% and 10% are observed from the tightest *idleslope* setting of other priority flows, such as 52 Mbps and 35 Mbps for *idleslope*_B_ settings at ports SF→SB and SB→SF under SR-A evaluation case, they might be a little pessimistic but should be safe for looser configurations. 

### 5.3. Shaping Benefit Explanation 

**Figure 10:**
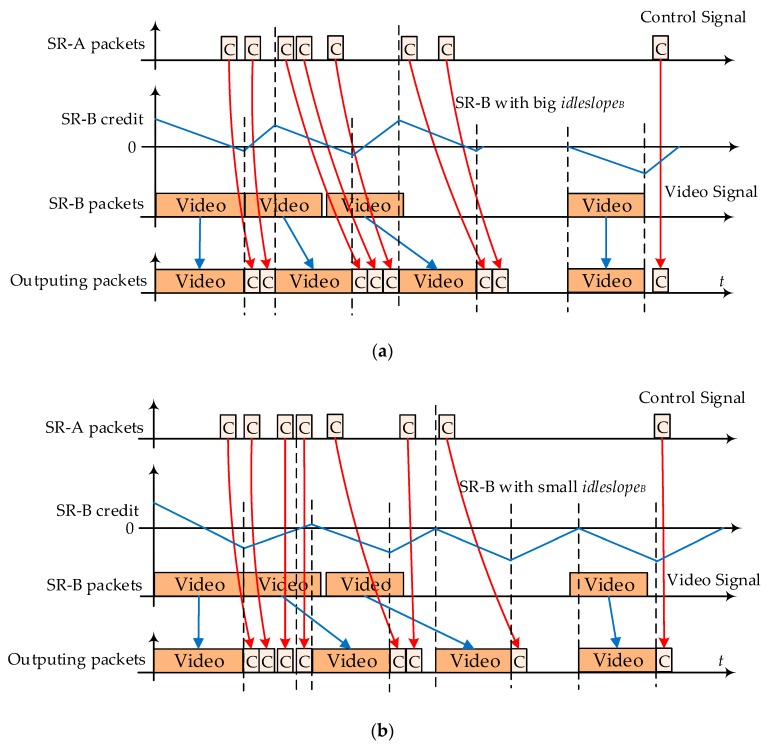
Interference among frames with different priorities: (**a**) SR-B with large *idleslope_B_*; and (**b**) SR-B with small *idleslope_B_*.

In Section 5.1, we found that the postponement by CBS shaping could bring benefits to other priority flows transmissions. The shaping benefit from SR-A to SR-B is easy to explain since a tighter *idleslope_A_* setting for SR-A often means more transmission intervals and opportunities for SR-B flows while SR-A flows are waiting credit recovery. However, the benefit from SB-B to SR-A is not so easy to figure out since SR-A flows are assigned with the highest priority and always have the priority to be sent out first. Figure 10 shows the interference among different flows and tries to explain the shaping benefit from low priority to high priority. Figure 10a shows the interference with a large *idleslope_B_* setting for SR-B flows results in heave blocking for SR-A, and Figure 10b shows the opposite situation: a small *idleslope_B_* setting for SR-B results in light blocking for SR-A.

We can observe that if SR-B is assigned with a larger *idleslope_B_*, its frames tend to be scheduled out more quickly after a shorter credit recovery interval. Maybe in some region, there are more SR-B frames concentrating together. Even though SR-A has the highest priority, its frames have to wait for the current frame to finish its transmission, and then its frames can grab their transmission chance. Therefore, within the SR-B concentrating region, there is a greater likelihood of blocking by SR-B frames to SR-A frames, and this kind of blocking might result in larger average delays of SR-A flows especially when SR-B flows are configured with big frame length, such as 1522 bytes of VS. On the contrary, if SR-B is assigned with a relatively smaller *idleslope_B_*, its frames tend to be scheduled out more slowly after a longer credit recovery interval, which means SR-B frames will be sent our more regularly with less flow bursts. Therefore, if several SR-B frames arrive together, these frames will be isolated by SR-B small *idleslope*. If SR-A frames happen to arrival in the same time region, they will suffer light blocking by SR-B frames since SR-B frames are separated from each other.

### 5.4. Further Discussion From Network Calculus 

In this section, we focus on CBS shaping benefit analysis from Network Calculus perspective. The consideration of using network calculus mainly comes from its convenience of modeling flow maximal burst. Indeed, a bigger *idleslope* setting could bring larger flow burst as a looser flow control mechanism. This kind of enlarging flow burst might be the potential factor for different shaping benefit. Firstly, we will carry on the study about flow maximal burst and propose a method to measure its equivalent logical maximum, which considers the actual arrival behavior of frames with serialization effect. Secondly, by using integral operation, the detailed shaping benefits are fitted according to the changing of flow burst.

#### 5.4.1. Maximal Burst Analysis

The basic concept of Network Calculus can be found in Appendix B. According to its theory, *R* is used to define one flow, and *R*(*t*) is the value of the flow at time *t*. When flow *R* is scheduled out from an output port after entering into the backbone of a switched network, its equal departure burst will become fuzzier and larger, and this kind of burst enlargement can lead to a further uncertain of flow’s arrival in the following switches. The enlargement burst can be calculated according to Equation (5) if flow *R* strictly obeys the leaky bucket flow model [39].

(5) $$\begin{array}{l} \alpha (t)=\sigma_{flow}^{in}+\rho \times t\\ \sigma_{flow}^{out}=\sigma_{flow}^{in}+\rho \times D\\ \alpha^{*}(t)=\sigma_{flow}^{out}+\rho \times t \end{array}$$

where α(*t*) is the arrival curve for flow *R* and α^*^(*t*) is the arrival curve for the corresponding output flow R^*^; $\sigma_{flow}^{in}$ is the burst on arrival and $\sigma_{flow}^{out}$ is the burst after departure; ρ is flow constant bit rate; and D is the worst-case delay of flow *R* in the current output port.

Furthermore, if a *shaper* is adopted to shape this flow, the corresponding output flow R^*^ would also be restricted within an envelope defined by the shaper during a period of time ∆*t*:

(6) $$R^{*}(t+\Delta t)-R^{*}(t)\leq shaper(\Delta t)$$

In other words, the departure burst is limited by the shaper and the arrival curve α^*^(*t*) for R^*^ is restricted by:

(7) $$\alpha^{*}(t)=\min\{\sigma_{flow}^{out}+\rho t,shaper(t)\}$$

Equation (7) shows the flow control mechanism by a shaper from the burst aspect. It limits the maximum flow burst for the next switch. In other words, if a shaper is applied to a flow, its burst will be limited within a scope, which perhaps could bring some benefits to other flows. By using Equation (7), the expression of α(*t*) and α^*^(*t*) as well as the worst-case delay at any output port along flow paths can be calculated.

The CBS algorithm in Ethernet-AVB plays the role in flow shaping. Considering the worst-case scheduling scenario for SR-A, the maximum block for SR-A is expected as the synchronous arrival of a lower priority frame with the longest frame length. Though SR-A has the highest priority, it cannot break the current frame transmission even with lower priority. In Figure 2, the first coming BE frame occupies the transmission chance in the physical link, then the next coming SR-A frames have to wait for the end of BE transmission. Thus, the maximum blocking time for SR-A is bounded by:

(8) $$T_{A}=\frac{l_{\max}^{n}}{C},\;l_{\max}^{n}=\max\{l_{\max}^{B},l_{\max}^{BE}\}$$

where $l_{\max}^{B}$ is the maximum frame length for SR-B, $l_{\max}^{BE}$ is the maximum frame length for BE and *C* is the link speed.

During the blocking time *T_A_*, SR-A gains the credit up to $Credit_{H}^{A}=T_{A}\times idleslope_{A}$ [30], which is the highest reachable credit for SR-A. The lowest reachable credit for SR-A is $Credit_{L}^{A}=l_{\max}^{A}/C\times sendslope_{A}$ [30] only when SR-A credit is just consumed from zero with the sending behavior of a SR-A frame assigned as the maximal frame length $l_{\max}^{A}$. Thus, the longest continuous transmission time for SR-A can be obtained. It depends on the time interval when SR-A credit decreases from the highest credit to the lowest one with a rate of *sendSlope_A_* as:

(9) $$T_{{}_{C_{H}->C_{L}}}^{A}=\frac{Credit_{H}^{A}-Credit_{L}^{A}}{-sendslope_{A}}$$

According to Equation (9), the maximum amount of bits that can be transmitted back-to-back for SR-A is:

(10) $$\begin{array}{ll} bit_{{}_{C_{H}->C_{L}}}^{A}=T_{{}_{C_{H}->C_{L}}}^{A}\times C & =\frac{idleslope_{A}}{-sendslope_{A}}\times l_{\max}^{n}+l_{\max}^{A}\\ & =\frac{idleslope_{A}}{C-idleslope_{A}}\times l_{\max}^{n}+l_{\max}^{A} \end{array}$$

Equation (10) gives the maximal continuous sending bits during the worst-case queuing scenario for SR-A, and it also matches with the result shown in [29], in which the author explains it is the maximal permitted burst tolerance for SR-A flows. 

In [30], the authors rethink the shaping process according to min-plus theory and deduce the detailed expressions for AVB SR shapers. For SR-A, the shaper curve [30] is:

(11) $$\sigma^{A}(t)=idleslope_{A}\times \left(t+\frac{l_{\max}^{n}}{C}+l_{\max}^{A}\times \frac{C-idleslope_{A}}{idleslope_{A}\times C}\right)$$

Letting *t* = 0 can obtain the initial maximal permitted burst $\sigma_{CBS-M}^{A}$ as:

(12) $$\begin{array}{l} \sigma_{CBS-M}^{A}=\sigma^{A}(t=0)\\ \Rightarrow \sigma_{CBS-M}^{A}=\frac{idleslope_{A}}{C}\times \left(l_{\max}^{n}-l_{\max}^{A}\right)+l_{\max}^{A} \end{array}$$

In other words, $\sigma_{CBS-M}^{A}$ is the initial value of the shaper curve for SR-A according to Equation (11) when time *t* is set to zero. In fact, it can also be got from $bit_{{}_{C_{H}->C_{L}}}^{A}$. If we consider the maximum amount of bits transmitted back-to-back could be the sum of the initial maximal permitted burst plus the bits which can be accumulated during the time interval of the maximal transmission windows, the initial maximal permitted burst $\sigma_{CBS-M}^{A}$ for SR-A can be got from:

(13) $$bit_{{}_{C_{H}->C_{L}}}^{A}=\sigma_{CBS-M}^{A}+idleslope_{A}\times T_{{}_{C_{H}->C_{L}}}^{A}$$

Therefore,

(14) $$\begin{array}{l} \sigma_{CBS-M}^{A}=bit_{{}_{C_{H}->C_{L}}}^{A}-idleslope_{A}\times T_{{}_{C_{H}->C_{L}}}^{A}=T_{{}_{C_{H}->C_{L}}}^{A}\times C-idleslope_{A}\times T_{{}_{C_{H}->C_{L}}}^{A}\\ \begin{array}{cc} & \;\;=-sendslope_{A}\times T_{{}_{C_{H}->C_{L}}}^{A}=Credit_{H}^{A}-Credit_{L}^{A} \end{array} \end{array}$$

According to the definitions of $Credit_{H}^{A}$ and $Credit_{L}^{A}$, $\sigma_{CBS-M}^{A}$ can be further deduced as:

$$\begin{array}{l} \sigma_{CBS-M}^{A}=\frac{idleslope_{A}}{C}\times l_{\max}^{n}-\frac{sendslope_{A}}{C}\times l_{\max}^{A}\\ \begin{array}{cc} & \;\;=\frac{idleslope_{A}}{C}\times \left(l_{\max}^{n}-l_{\max}^{A}\right)+l_{\max}^{A} \end{array} \end{array}$$

**Figure 11:**
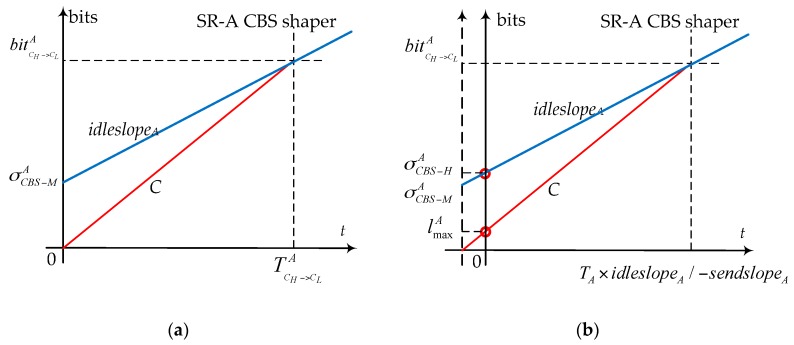
The illustration about the maximal permitted burst defined by SR-A CBS shaper: (**a**) the relationship between $bit_{{}_{C_{H}->C_{L}}}^{A}$ and $\sigma_{CBS-M}^{A}$; and (**b**) the relationship between $\sigma_{CBS-M}^{A}$ and $\sigma_{CBS-H}^{A}$.

Thus, the initial maximal permitted burst $\sigma_{CBS-M}^{A}$ according to Equation (11) in [30] can also be obtained from the maximum amount of bits $bit_{{}_{C_{H}->C_{L}}}^{A}$. Figure 11a shows the relationship between $bit_{{}_{C_{H}->C_{L}}}^{A}$ and $\sigma_{CBS-M}^{A}$. 

Equation (12) gives the initial maximal permitted burst according to the definition of shaping function from min-plus theory perspective, but it does not consider the actual arrival behavior of frames, as well as the serialization effect which has been used to tighten the upper bound of flow end-to-end delay typically. In fact, the flow with the maximal frame length constitutes the initial maximum burst among the flows sharing the same physical link when they flow into the next switch together. Adopting a similar method mentioned in [37], the arrival curve for the coming serialized flows (grouped flows) should begin from one maximal frame length with the rate as the link speed, then it will be restricted by the CBS logical bandwidth; at last, it keeps increasing according to its long term constant bit rate. For the CBS shaper, the shaping envelope should also start from the corresponding one maximal frame length. Under this consideration, we can define a new burst $\sigma_{CBS-H}^{A}$ to descript this kind of shaping features. $\sigma_{CBS-H}^{A}$ is used to represent the equivalent maximum burst of SR-A, which considers the fact that the frame with the maximal frame length has already arrived at the output port and been selected out to cause the initial maximum burst. Thus, the following accumulated maximal flow bits should be less than $bit_{{}_{C_{H}->C_{L}}}^{A}$. Then, we can get $\sigma_{CBS-H}^{A}$ as:

(15) $$\begin{array}{l} \sigma_{CBS-H}^{A}+idleslope_{A}\times \frac{bit_{{}_{C_{H}->C_{L}}}^{A}-l_{\max}^{A}}{C}=bit_{{}_{C_{H}->C_{L}}}^{A}\\ \Rightarrow \sigma_{CBS-H}^{A}=\frac{idleslope_{A}}{C}\times l_{\max}^{n}+l_{\max}^{A} \end{array}$$

In Equation (15), the subtraction of $l_{\max}^{A}$ from $bit_{{}_{C_{H}->C_{L}}}^{A}$ considers $l_{\max}^{A}$ has arrived at the output port. Compared to $bit_{{}_{C_{H}->C_{L}}}^{A}$ and $\sigma_{CBS-M}^{A}$, our $\sigma_{CBS-H}^{A}$ considers the actual arrival behavior of frames with serialization effect and shows the equivalent logical maximum of bursts. 

According to the subtraction operation in Equation (15), the shaping envelope with serialization effect for SR-A CBS can also be obtained by moving the ordinate in Figure 9a a specific length to the right as $l_{\max}^{A}/C$, which is shown in Figure 11b. The initial value of the moved shaper curve is our equivalent maximum burst $\sigma_{CBS-H}^{A}$. In Figure 11b, the *C* speed line starts from $l_{\max}^{A}$. The time of the crossing point between *C* speed line and *idleslope_A_* line is $T_{{}_{C_{H}->C_{L}}}^{A}-l_{\max}^{A}/C=T_{A}\times idleslope_{A}/-sendslope_{A}$ since it already has one frame ready. 

Focused on SR-B CBS shaping function, we can adopt the same way to obtain the maximum amount of bits transmitted back-to-back $bit_{{}_{C_{H}->C_{L}}}^{B}$, the maximal permitted burst $\sigma_{CBS-M}^{B}$ and the equivalent maximal burst $\sigma_{CBS-H}^{B}$. To achieve this purpose, the maximum blocking time for SR-B should be calculated first, which seriously depends on the worst-case scheduling scenario. As the priority of SR-A is higher than SR-B, the longest burst transmission of SR-A flows is expected to constitute the worst-case scheduling scenario for SR-B flows. Thus, for SR-B the worst-case blocking occurs at the end of the transmissions of a BE frame plus the possibly maximum numbers of SR-A frames with the corresponding maximal frame length, so it is bounded by:

(16) $$T_{B}=\frac{l_{\max}^{BE}}{C}+T_{{}_{C_{H}->C_{L}}}^{A}=\frac{l_{\max}^{A}}{C}+\frac{l_{\max}^{BE}}{C}+\frac{idleslope_{A}}{C-idleslope_{A}}\times \frac{l_{\max}^{n}}{C}$$

The highest and lowest reachable credits for SR-B can be got according to the same way for SR-A analysis, which are $Credit_{H}^{B}=T_{B}\times idleslope_{B}$ and $Credit_{L}^{B}=l_{\max}^{B}/C\times sendslope_{B}$. With these results, the longest continuous transmission time for SR-B can also be obtained.

(17) $$T_{{}_{C_{H}->C_{L}}}^{B}=\frac{Credit_{H}^{B}-Credit_{L}^{B}}{-sendslope_{B}}$$

Thus, the maximum amount of bits that can be transmitted back-to-back for SR-B is:

(18) $$\begin{array}{l} bit_{{}_{C_{H}->C_{L}}}^{B}=T_{{}_{C_{H}->C_{L}}}^{B}\times C=\frac{Credit_{H}^{B}-Credit_{L}^{B}}{-sendslope_{B}}\times C=\frac{T_{B}\times idleslope_{B}-\frac{l_{\max}^{B}}{C}\times sendslope_{B}}{-sendslope_{B}}\times C\\ \Rightarrow bit_{{}_{C_{H}->C_{L}}}^{B}=\left(l_{\max}^{BE}+l_{\max}^{A}+\frac{idleslope_{A}}{C-idleslope_{A}}\times l_{\max}^{n}\right)\times \frac{idleslope_{B}}{C-idleslope_{B}}+l_{\max}^{B} \end{array}$$

For the initially maximal permitted burst $\sigma_{CBS-M}^{B}$, it obeys a similar restriction, as shown in Equation (13), and can be further calculated as:

(19) $$\begin{array}{l} \sigma_{CBS-M}^{B}=bit_{{}_{C_{H}->C_{L}}}^{B}-idleslope_{B}\times T_{{}_{C_{H}->C_{L}}}^{B}=Credit_{H}^{B}-Credit_{L}^{B}\\ \begin{array}{cc} & \;\;=\left(l_{\max}^{BE}+l_{\max}^{A}+\frac{idleslope_{A}}{C-idleslope_{A}}\times l_{\max}^{n}-l_{\max}^{B}\right)\times \frac{idleslope_{B}}{C}+l_{\max}^{B} \end{array} \end{array}$$

With the expression of $bit_{{}_{C_{H}->C_{L}}}^{B}$, our equivalent maximal burst $\sigma_{CBS-H}^{B}$ can be deduced out. It also considers the fact that the initial maximum burst of SR-B comes from the frame with the maximal frame length.

(20) $$\begin{array}{l} \sigma_{CBS-H}^{B}+idleslope_{B}\times \frac{bit_{{}_{C_{H}->C_{L}}}^{B}-l_{\max}^{B}}{C}=bit_{{}_{C_{H}->C_{L}}}^{B}\\ \Rightarrow \sigma_{CBS-H}^{B}=\left(l_{\max}^{BE}+l_{\max}^{A}+\frac{idleslope_{A}}{C-idleslope_{A}}\times l_{\max}^{n}\right)\times \frac{idleslope_{B}}{C}+l_{\max}^{B} \end{array}$$

#### 5.4.2. Shaping Benefit Computing

According to the discussion above, the burst restriction by CBS shapers could bring benefit to other priority flows as a tighter burst restriction often means more transmission opportunities for others. Besides that, the extra bandwidth reserving than the strictly necessary also plays a role on the final benefit. In fact, it can be seen as an amplifier during the benefit computing.

Considering an actual output port with CBS algorithm, different priority flows share the common bandwidth, such as *C* = 100 Mbps, with different *idleslope* settings. Focused on the shaping benefit from SR-A to SR-B, the maximum amount of bits that are transmitted back-to-back for SR-A can all be responsible for the delay to SR-B flows since it has the highest priority and constitutes the actually maximal blocking for SR-B flows. If the *idleslope_A_* setting varies, the change of the maximum transmission amount can be calculated according to Equation (10), such as the decrease of *idleslope_A_* from 10 Mbps to 5 Mbps at the port from SF to SB can result in a burst reduction as 90.18 bytes. Supposing the shaping benefit mainly comes from the burst reduction of SR-A, it can be calculated as:

(21) $$\Delta bit_{{}_{C_{H}->C_{L}}}^{A}\times \frac{8}{C}=90.18\;\mathrm{bytes}\;\times \;8\;\mathrm{bit}\;/\;100\mathrm{Mbps}\;=\;7.2\;\mu s$$

The result shown in Equation (21) is quite rough since it only considers the change of the maximum burst. A more accurate computing method should investigate the cumulative process of shaping benefit during the varying scope of *idleslope_A_* setting.

According to Equation (10), any little variation of *idleslope_A_* could cause a burst change. It can be approached from the first-order derivative of Equation (10) as:

(22) $$\Delta bit_{{}_{C_{H}->C_{L}}}^{A}(\Delta idleslope_{A})={\left(bit_{{}_{C_{H}->C_{L}}}^{A}\right)}^{\prime }\times \Delta idleslope_{A}=\frac{C\times l_{\max}^{n}}{{\left(C-idleslope_{A}\right)}^{2}}\times \Delta idleslope_{A}$$

When $\Delta bit_{{}_{C_{H}->C_{L}}}^{A}(\Delta idleslope_{A})$ is used to calculate the shaping benefit, it will be amplified by bandwidth reservation margin of SR-A. The amplifying computing is based on the scale effect of bandwidth. A bigger bandwidth margin always means larger space for SR-A CBS performs. Letting *band_A_* represent the actual bandwidth occupation by SR-A flows, the bandwidth margin can be normalized as $(idleslope_{A}-band_{A})/C$. Supposing the shaping benefit from SR-A is positively associated with its bandwidth margin, any little burst change of SR-A finally results in a benefit to SR-B as:

(23) $$\Delta benefit_{A->B}=\Delta bit_{{}_{C_{H}->C_{L}}}^{A}(\Delta idleslope_{A})\times \frac{idleslope_{A}-band_{A}}{C}\times \frac{8}{C}\times K$$

where *K* is a balance coefficient with an expectation value as $K=\exp\left(C/band_{all}-1\right)$ and 8/C is to change the burst into delay time. In the expression of *K*, *band_All_* represents the total bandwidth occupation by all priority flows, including SR-A flows, SR-B flows and BE flows. Parameter *K* reflects the bandwidth margin for all flows. Similar as the scale effect of SR-A bandwidth margin, a bigger value of *K* always means a larger margin for all flows, and it would permit more space for CBS shaping function performing, then finally, it could potentially bring a bigger benefit expectation. The exponentiation operation indicates its linear strength during the variation of *band_All_*.

Compared to the calculation method shown in Equation (21), Equation (23) gives the differential form of shaping benefit from SR-A and counts the effect of bandwidth margin. Through integral operation, the detailed value of benefit can be got as:

(24) $$\begin{array}{l} benefit_{A->B}=\int \frac{\Delta benefit_{A->B}}{\Delta idleslope_{A}}\times d(idleslope_{A})\\ \begin{array}{c} \;\;\;=\frac{8}{C}\times \int_{idleslope_{A1}}^{idleslope_{A2}}{\left(bit_{{}_{C_{H}->C_{L}}}^{A}\right)}^{\prime }\times \frac{idleslope_{A}-band_{A}}{C}\times d(idleslope_{A})\times K \end{array}\\ \begin{array}{c} \;\;\;= \end{array}\frac{8\times l_{\max}^{n}}{C}\times K\times {\left[\ln\left(C-idleslope_{A}\right)+\frac{C-band_{A}}{C-idleslope_{A}}\right]}_{idleslope_{A1}}^{idleslope_{A2}} \end{array}$$

For the shaping benefit from SR-B to SR-A, the maximum amount of SR-B flows that are transmitted back-to-back cannot all be used to postpone the transmission of SR-A flows. However, a large amount of continuous SR-B flows have great possibility to cause uneven concentrating regions, and this regions can be logically measured by the equivalent logical maximum of bursts as $\sigma_{CBS-H}^{B}$, which could finally affect the transmission of high priority flows. Adopting the same way for SR-A shaping benefit analysis, the differential form of SR-B equivalent logical maximum of bursts is:

(25) $$\begin{array}{l} \Delta \sigma_{CBS-H}^{B}(\Delta idleslope_{B})={\left(\sigma_{CBS-H}^{B}\right)}^{\prime }\times \Delta idleslope_{B}\\ \;\;\;\;\;\;\;\;=\frac{l_{\max}^{BE}+l_{\max}^{A}+\frac{idleslope_{A}}{C-idleslope_{A}}\times l_{\max}^{n}}{C}\times \Delta idleslope_{B} \end{array}$$

Thus, the shaping benefit from SR-B to SR-A is:

(26) $$\begin{array}{l} benefit_{B->A}=\frac{8}{C}\times \int_{idleslope_{B1}}^{idleslope_{B2}}{\left(\sigma_{CBS-H}^{B}\right)}^{\prime }\times \frac{idleslope_{B}-band_{B}}{C}\times d(idleslope_{B})\times K\\ \begin{array}{c} \;\;\;= \end{array}\frac{8\times \left(l_{\max}^{BE}+l_{\max}^{A}+\frac{idleslope_{A}}{C-idleslope_{A}}\times l_{\max}^{n}\right)}{C^{3}}\times K\times {\left[\frac{{\left(idleslope_{B}-band_{B}\right)}^{2}}{2}\right]}_{idleslope_{B1}}^{idleslope_{B2}} \end{array}$$

In Equation (26), *band_B_* is used to represent the actual bandwidth occupation by SR-B flows. According to Equations (24) and (26), the shaping benefits from CBS can be calculated and the results are shown in Table 5 and Table 6. Table 5 shows the shaping benefits from SR-A to SR-B according to Equation (24), and Table 6 shows the shaping benefits from SR-B to SR-A according to Equation (26). For contrast, the simulation results are also given in Table 5 and Table 6. These simulation results come from the statistical data shown in Figure 9 in Section 5.1. All these results are based on the average delay, not the maximal one. For example, for flow CS_RearC_→TV under SR-A evaluation case in Figure 9b, the reduction value of delays between the curve of *idleslope*_B_(SB→SF) = 65 Mbps and curve of *idleslope*_B_(SB→SF) = 35 Mbps at point of 25 Mbps for *idleslope*_A_(SB→SF) is 15 µs. This difference only comes from the different settings of *idleslope*_B_, thus, it can be seen as the shaping benefit from SR-B to SR-A. For contrast, the calculated benefit is 12.2 µs by Equation (26).

According to the data in Table 5 and Table 6, the calculated shaping benefits match well with the simulation results. For flow VS_RearC_→HU and VS_RearC_→TV, the shaping benefits from SR-A are no more than 2 µs according to the simulation experiments, and the analytic values are also limited into 2 µs. If the priorities of CS flows and VS flows are exchanged, the analytic values still follow the simulation results and reflect the changing of the maximal burst. Focused on the shaping benefits from SR-B, the analytic method also achieves a good prediction effect.

Besides, it is a good way for the analytic method to explain the varying trend of CBS shaping benefits. According to Equations (10) and (24), the maximal transmitted bits amount $bit_{{}_{C_{H}->C_{L}}}^{A}$ and the shaping benefit $benefit_{A->B}$ have no direct relationship with SR-B *idleslope_B_*. Thus, the shaping benefits for flow CS_FC_→TV (priority swapped) at different *idleslope_B_* settings from *idleslope_A_* = 65 Mbps to *idleslope_A_* = 55 Mbps nearly keep the same, which are shown in Figure 9e. In addition, the differences among different shaping benefits for flow CS_RearC_→HU (priority swapped) at different *idleslope_B_* settings between the curve of *idleslope_A_* = 65 Mbps and the curve of *idleslope_A_* = 35 Mbps basically hold a similar gap shown in Figure 9f. This kind of varying trend is quite different from the shaping benefit from SR-B to SR-A, as shown in Figure 9a,b. We can observe that the benefit differences get bigger with the increase of *idleslope_A_* setting. In fact, both the equivalent logical maximum of bursts $\sigma_{CBS-H}^{B}$ and the shaping benefit $benefit_{B->A}$ are directly related with *idleslope_A_* setting according to Equations (20) and (26). A bigger *idleslope_A_* will bring a larger $\sigma_{CBS-H}^{B}$ and consequently results in a bigger burst incremental and this incremental finally constitutes the increasing gap trend among different *idleslope_B_* curves. 

## 6. Discussions 

According to the analysis of shaping restriction and shaping benefit, as well as the effects of CBS logical bandwidth allocation, we can get some suggestions for CBS parameters setting.

Firstly, shaping restriction by CBS dominates flow average delays since it determines the maximal logical bandwidth. In Section 5.2, two margin degrees are given: one is 1.7 for 10% gain threshold, and the other is 2.7 for 5% gain threshold. Bandwidth reservation margin degree can be obtained from Equation (3) and the gain from shaping restriction can be got from Equation (4). If we want to accelerate a certain SR class flows transmission, the simplest way is to increase its *idleslope* setting. However, if the margin degree is higher than 1.7 or even higher than 2.7, the corresponding gain from a looser shaping restriction is lower. For example, if we want to decrease the average delay by 1% with the allocated margin degree as 2.7, the corresponding bandwidth reservation margin should be expanded by 20% on the base of the current setting. With this configuration, the endeavor to cut down flow average delays is inefficient. Besides, the allocation of bandwidths for multi-priority CBSs should consider the overall performance requirements, especially when resources in industrial systems are limited. Under this circumstance, the gain for each type of SR class should be considered twice to find an optimal balance among different *idleslope* settings. 

By using 1.7 for 10% gain and 2.7 for 5% gain, we can find most of the original *idleslope* settings are reasonable. For example, the margin degrees at end system FC are 13.9 and 2.8 for SR-A and SR-B respectively. Even if a larger *idleslope* was assigned to these CBSs, the effectiveness of reducing average delays is small. For port SF→SB, the corresponding margin degrees are 0.86 for SR-A and 0.26 for SR-B. Thus, SR-B flows receive much stricter shaping restriction than SR-A flows. In fact, the original *idleslope*_B_ at port SF→SB could be set to 70 Mbps, which can increase the margin degree from 0.26 to 0.36, which also will reduce the average delays for SR-B flows. For port SB→SF, the corresponding margin degrees are 0.86 for SR-A and 0.89 for SR-B, in other words, SR-A flows and SR-B flows receive a similar flow control pressure. If the system designer wants to accelerate the transmission for SR-A flows, the remaining 5 Mbps logical bandwidth can be added into *idleslope*_A_, otherwise, added into *idleslope*_B._ The remaining 5 Mbps bandwidth comes from the assumption that the sum of the reserved bandwidths for SR-A and SR-B flows is recommended not to exceed 75% of the physical bandwidth. Of course, another allocation plan for the remaining 5 Mbps might be 1 Mbps for SR-A flows and 4 Mbps for SR-B flows or so on. Thus, it can consider the two sides to fully utilize the high gains (18.0% for SR-A and 23.1% for SR-B) from shaping restriction. Of course, if the sum of SR-A and SR-B reserved bandwidths can be assigned to a higher threshold, such as 90%, the end-to-end delays can be decreased more remarkably. 

Secondly, shaping benefit from other CBSs can partly affect the final flow average delays. In Section 5.4, shaping benefit has been modeled and calculated according to the changing of flow maximal burst or the equivalent logical maximum of bursts. The detailed computing methods are shown in Equation (24) for the benefit from SR-A to SR-B and Equation (26) for the benefit from SR-B to SR-A. For a given SR class, as well as its allocated logical bandwidth, the average delays of flows belonging to this priority class still can be affected by other priority CBS settings. Generally speaking, a tighter *idleslope* setting of other priority CBSs could bring more shaping benefit to the considered flows. If we want to continue to cut down flows average delays without adjusting their own *idleslope*, the only way is to decrease other priority CBS *idleslope* settings. For example, according to the analysis result for the benefit from SR-B to SR-A, the decreasing of *idleslope*_B_ from 65 Mbps to 45 Mbps at port SB→SF could bring 10.7 μs benefit for the average delay of flow CS_RearC_→HU when its own *idleslope*_A_ is 25 Mbps. However, if *idleslope*_A_ is 5 Mbps, the same decreasing range of *idleslope*_B_ can only achieve 3.0 μs benefit. We can also adopt the same way to reduce the average delays for SR-B flows based on the analysis result for the benefit from SR-A to SR-B. However, using shaping benefit to improve flow delays can only be seen as an alternative method to solve CBS logical bandwidth allocation problem. A more appropriate application of it lies in the analysis for delay difference at a given *idleslope* setting, which is what we have done in Section 5.4.2.

According to the discussion above, the final bandwidth allocation for CBS in Ethernet-AVB lies in the balance of shaping restriction and shaping benefit. In addition, it should consider the balance among different priority CBS *idleslope* settings as the total bandwidth providing is always limited.

## 7. Conclusions 

In this paper, Ethernet-AVB, as well as its further proposal TSN/TAS, and AFDX are compared by simulation on a representative automotive case study. In order to match the flows defined in this case, the standard CMI in Ethernet-AVB is changed into flow’s period, and several VLs in AFDX are adopted to simulate one AVB flow.

According to our simulation results, the transmission of SR flows in Ethernet-AVB is shaped by its own *idleslope* setting which mainly determines flows queuing latencies as well as the final end-to-end delays, but also the transmission can benefit from the shaping operation by other priority CBSs. The final delays depend on the balance of their own shaping restriction and other priority CBS shaping benefits. AFDX has no such a shaping mechanism and is lack of a further method for flow controlling. 

As expected, simulation results show that AFDX gives smaller delay for high priority flows and larger delays for low priority ones on the considered case. The most interesting part concerns the medium priority flows. There is no clear winner, since it depends on flow and AVB shaper configurations. The Ethernet-AVB shaping for SR-A flows mitigates the impact of SR-A frame length on SR-B flows. Thus, a high load of SR-A flows tends a better performance (both the average delay and the maximal delay) for SR-B flows in Ethernet-AVB than that for LP flows in AFDX. When TSN/TAS is considered, the transmission delay and jitter for the highest priority flows (CDT) can be fully guaranteed by synthesizing of time-aware transmission gates. However, different arrangements of transmission gates will bring different end-to-end delays both for the highest priority CDT flows and other priority flows, such as SR flows and BE flows.

According to network calculus theory, the CBS shaping benefit can be partly explained. A tighten *idleslope* setting always means less flow bursts during the shaping process, which will decrease the degree of burst uncertain and expansion for flow arrival curves. This trend will increase the forwarding opportunities for other priority flows, and finally bring some shaping benefits to others. By using the analytic method, the shaping benefit is related to the change of the maximal burst; moreover, it has relationship with the bandwidth reservation margin. A bigger burst change and a larger bandwidth reservation can result in more shaping benefits to other priority flows. It suits the benefit analysis from SR-A flows to SR-B flows, as well as suits the analysis from SR-B flows to SR-A flows. 

Combining the analysis of shaping restriction and shaping benefit, some suggestions on CBS logical bandwidth allocation have been given. With the help of SR bandwidth reservation margin degree, two thresholds can be used to guide the setting for CBS *idleslope*: if the margin degree is 1.7, the gain from shaping restriction is 10%, and the margin degree is 2.7, the gain is 5%. However, still delays of flows can be affected by the bandwidth allocation settings of other priority CBSs. The detailed improvement by decreasing other priority CBS *idleslope* setting can be estimated according to the proposed analytical method. 

Future work includes the upper bound analysis of the worst-case delay and comparison for AVB and AFDX, as well as for TSN.

## Figures and Tables

**Table 1:** Flow parameters for the industrial case.

Signal	Frame Length ^1^ (bytes)	Priority (AVB, AFDX, TAS)	MIF	CMI (ms)	Bandwidth (Mbps)
Control Signal	64	SR_A, HP, CDT	10	10	0.67
Video Signal	1522	SR_B, LP, SR-B	46	33	17.2
MVideo Signal	1522	BE, BE, BE	43	33.33	15.9

^1^ Frame length does not include the interframe gaps (12 bytes) and preambles (1 + 7 bytes).

**Table 2:** Comparison of end-to-end delay.

VL	Average Delay	Maximal Delay	Real Worst-Case Delay [37]
v1	152.76	267.47	272
v2	152.42	190.12	192
v3	153.10	254.06	272
v4	152.82	271.10	272
v5	96.57	160.15	176

**Table 3:** Average delay and maximal delay for some example flows.

Signal	Avg Delay in AVB	Max Delay in AVB	Ratio ^1^ in AVB	Avg Delay in AFDX	Max Delay in AFDX	Ratio ^1^ in AFDX
CS_FC_→RC	32.1	236	7.35	30.4	149	4.90
CS_FC_→CU	134.1	912	6.80	93.1	285	3.02
CS_FC_→TV	332.6	1812	5.45	156.9	402	2.56
CS_RU_→CU	19.9	42	2.11	19.8	33	1.67
VS_LC_→TV	1520.6	5426	3.57	573.8	2083	3.63
VS_TV_→HU	488.6	2231	4.57	419.5	1346	3.21
MVS_HU_→RU	519	3383	6.52	666	4507	6.77
Min ratio ^1,2^	---	---	2.11	---	---	1.67
Max ratio ^1,2^	---	---	8.69	---	---	6.77
Ave ratio ^1,2^	---	---	6.29	---	---	3.70

^1^ Ratio = maximal delay/average delay; ^2^ the Min ratio, Max ratio and Ave ratio are collected from all flows.

**Table 4:** Gain of CBS shaping restriction for different *idleslope* settings (*idleslope* unit: Mbps).

**CS_FC_→TV under SR-A (SF→SB)**				**CS_RearC_→HU under SR-A (SB→SF)**			
***idleslope*_A_**	**Margin deg ^1^**	***idleslope*_B_**	***grad_CBS_***	***idleslope*_A_**	**Margin deg ^1^**	***idleslope*_B_**	***grad_CBS_***
5	0.86	52	20.6%	5	0.86	35	24.1%
		65	19.9%			65	18.0%
6	1.23	52	16.9%	6	1.23	35	19.9%
		65	16.6%			65	14.7%
7	1.60	52	14.9%	7	1.60	35	15.8%
		65	13.7%			65	11.9%
7.5	1.79	52	8.9%	7.5	1.79	35	9.9%
		65	8.4%			65	6.9%
10	2.72	52	5.0%	10	2.72	35	6.0%
		65	4.3%			65	3.7%
12.5	3.65	52	3.5%	12.5	3.65	35	4.1%
		65	3.2%			65	2.6%
15	4.58	52	1.9%	15	4.58	35	2.4%
		65	-			65	1.5%
**VS_RearC_→HU under SR-B (SB→SF)**				**VS_RearC_→TV under SR-B (SB→TV)**			
***idleslope*_B_**	**Margin deg ^1^**	***idleslope*_A_**	***grad_CBS_***	***idleslope*_B_**	**Margin deg ^1^**	***idleslope*_A_**	***grad_CBS_***
65	0.89	5	23.1%	70	0.02	5	9.7%
		10	23.1%			10	9.6%
70	1.03	5	20.2%	75	0.09	5	31.0%
		10	20.2%			10	31.1%
75	1.18	5	16.4%	80	0.16	5	35.1%
		10	18.2%			10	35.1%
80	1.33	5	16.7%	85	0.24	5	31.4%
		10	16.7%			10	31.4%
85	1.47	5	14.2%	90	0.31	5	22.1%
		10	14.2%			10	---
90	1.62	5	7.9%				
		10	-				
**CS_FC_→TV under SR-A (priority swapped) (SF→SB)**				**CS_RearC_→HU under SR-A (priority swapped) (SB→SF)**			
***idleslope*_B_**	**Margin deg ^1^**	***idleslope*_A_**	***grad_CBS_***	***idleslope*_B_**	**Margin deg ^1^**	***idleslope*_A_**	***grad_CBS_***
5	0.86	55	17.9%	5	0.86	35	23.0%
		65	16.6%			65	17.9%
6	1.23	55	14.1%	6	1.23	35	15.0%
		65	13.2%			65	15.9%
7	1.60	55	12.3%	7	1.60	35	23.5%
		65	10.7%			65	10.8%
7.5	1.79	55	7.2%	7.5	1.79	35	8.1%
		65	6.3%			65	7.4%
10	2.72	55	3.7%	10	2.72	35	6.4%
		65	3.2%			65	3.9%
12.5	3.65	55	2.8%	12.5	3.65	35	2.9%
		65	2.2%			65	2.6%
12.5	4.58	55	1.5%	15	4.58	35	3.7%
		65	-			65	0.0%

^1^ margin deg = bandwidth reservation margin/strict bandwidth requirement.

**Table 5:** CBS shaping benefit calculation from SR-A to SR-B.

Signal	Shaping Benefit (μs)	Simulation Result (μs)	*Port*	*K* ^1^	*idleslope_A2_* (Mbps)	*idleslope_A1_* (Mbps)	*idleslope_B_* (Mbps)
VS_RearC_→HU	1.9	<2	SB→SF	5.45	10	5	65
	1.9	<2			10	5	75
VS_RearC_→TV	0.3	<1	SB→TV	1.43	10	5	70
	0.3	<1			10	5	80
CS_FC_→TV ^2^	10.6	12	SF→SB	1.53	65	55	15
	10.6	11			65	55	5
CS_RearC_→HU ^2^	9.0	16	SB→SF	5.45	65	35	25
	9.0	10			65	35	5

**^1^** *K* considers the interframe gaps (12 bytes) and preambles (1 + 7 bytes) for all flows. ^2^ CS flows are assigned with SR-B priority when all CS and VS flows priorities are swapped.

**Table 6:** CBS shaping benefit calculation from SR-B to SR-A

Signal	Shaping Benefit (us)	Simulation Result (us)	*Port*	*K* ^1^	*idleslope_B2_* (Mbps)	*idleslope_B1_* (Mbps)	*idleslope_A_* (Mbps)
CS_FC_→TV	2.1	2	SF→SB	1.53	65	52	15
	1.9	1			65	52	5
CS_RearC_→HU	10.7	9	SB→SF	5.45	65	45	25
	12.2	15			65	35	25
	3.0	3			65	45	5
	3.4	6			65	35	5

^1^ *K* considers the interframe gaps (12 bytes) and preambles (1 + 7 bytes) for all flows.

**Figure A1:**
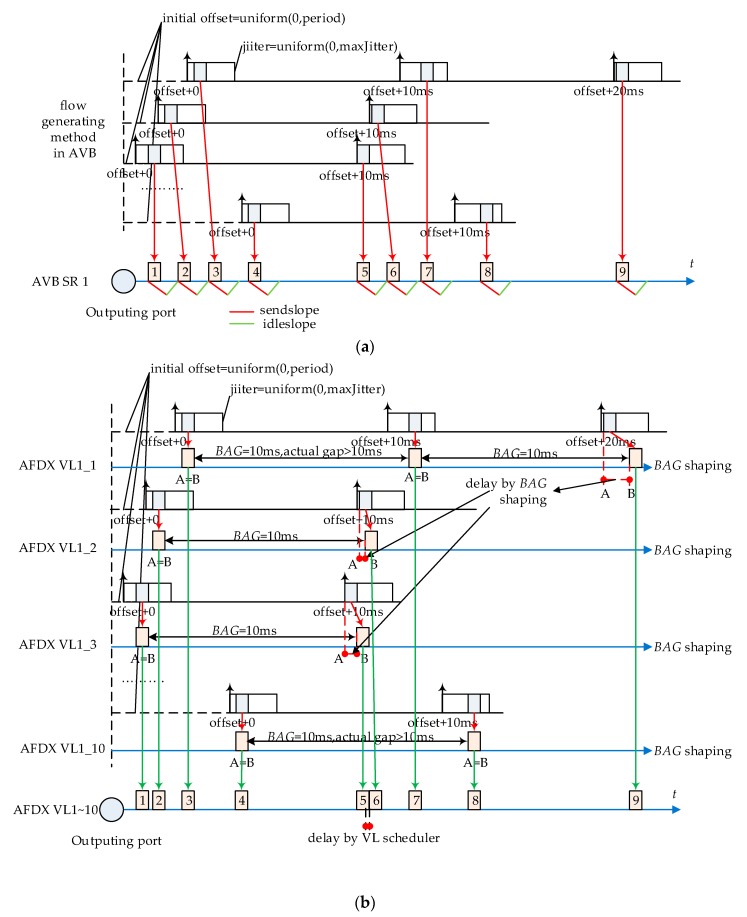
Flows generating methods for SR and rate constraint flows (taking Control Signal for example): (**a**) generating method in Ethernet-AVB; and (**b**) generating method in AFDX.

## Appendix A. The Generating Method of Flows in AVB and AFDX

The ARINC 653 [40] specification defines a partition scheduling strategy for avionics applications. Applications (or tasks) are executed in the pre-allocated time slops periodically in a Major Time Frame (MTF). Messages will be generated after the execution of applications, which determines the periods of messages consequently. In this paper, some common parameters for flows are considered: *offset*, *jitter* and *period*. Every flow has its own initial offset smaller than its period in most cases. For the sake of simplicity, a uniform distribution is adopted to generate the initial offset for each flow. In other words, its value is assigned as: *initial offset* = *uniform* (*0*, *period*). During the following simulating process, parameter *jitter* is used to reflect the uncertainty of flow generating window. The possibly maximal uncertain window is *maxJitter*. The detailed jitter for each instant of flow also follows a uniform distribution as: *jitter* = *uniform* (*0*, *maxJitter*). All flows are periodically generated with a constant payload. BE flows will be treated as the background traffic. To simplify its generating model, each BE flow is generated according to an exponential destitution with the mean value equal to *CMI*/*MIF*. For AVB SR flows and AFDX rate constraint flows (high priority flows and low priority flows), their generating methods are shown in Figure A1.

Figure A1 only gives an example to demonstrate the generating methods for Ethernet-AVB SR flows and AFDX rate constraint flows. One Control Signal is selected out to show the detailed simulation process. Ten VLs (from VL1_1 to VL1_10) in AFDX are used to imitate one CS flow in Ethernet-AVB. All flows are generated periodically with jitters, and the initial offsets determine the start time for them. The CS flow in Ethernet-AVB is shaped by its *idleslope* and *sendslope* settings at the output port. We can find the delays by Ethernet-AVB CBS function at the outputting line in Figure A1a, such as frames 2, 3 and 6. For the other frames (frames 1, 4, 5, 7, 8 and 9), they can be transmitted out immediately with an adequate remaining credit. For AFDX rate constraint flows, we can also observe the delays by VL *BAG* shaping function in Figure A1b. If the gaps between two consecutive frames carried by the same VL are less than the *BAG*, the follow-up frame will be postponed, such as frames 5, 6 and 9. After the shaping for all frames, they will be scheduled by a local VL scheduler, and it may cause further delays among different VLs, such as frame 6, which is delayed by frame 5 though it has been shaped by its own VL *BAG*. If there are multi-AVB streams, flow interferences will be quite complex, but the flows generating methods keep the same.

Two methods to measure the end-to-end delay in AFDX are shown in Figure A1b. One is named the A-method, and the other is named the B-method. The beginning time for A-method starts from the point of frame generating, which is denoted as “A” (before *BAG* shaper) in Figure A1b. For B-method, the beginning time starts from the point that the frame is just in transmission after *BAG* shaping, which is denoted as “B” (after *BAG* shaper). Thus, A-method counts the delays by *BAG* shapers. Generally speaking, the end-to-end delay of a flow in AFDX under A-method must be larger than that adopting B-method except one condition that the frame is not postponed by its *BAG* shaper because of a big enough arrival interval between the two adjacent frames, such as for frames 7 and 8 in Figure A1b, the beginning time for A-method and B-method is the same (A = B); However, for frames 5, 6, 9 in Figure A1b, A-method will get a bigger measurement result than B-method. Furthermore, the bigger the generating jitter is, the larger the difference between A-method and B-method should be. In this paper, we adopt the B-method to measure end-to-end delays in AFDX.

## Appendix B. The Basic Concept of Network Calculus

Network Calculus (NC) is a theoretical framework that provides deep insights into flow problems encountered in networking, especially for the problem of computing the end-to-end delays. Network Calculus is mathematically based on the Min-Plus dioid (also called Min-Plus algebra) [41], for which the convolution ⊗ and deconvolution ∅ operations between two functions *f*(*t*) and *g*(*t*) are defined by:

$$\begin{array}{l} (f⊗g)(t)=\underset{0\leq s\leq t}{\inf}\left\{f(t-s)+g(s)\right\}\\ (f\emptyset g)(t)=\underset{0\leq s}{\sup}\left\{f(t+s)-g(s)\right\} \end{array}$$

The basic concepts in Network Calculus are arrival curve and service curve. According to its theory, a flow *R* has an arrival curve *α*(*t*) if R(t)≤(R⊗α)(t) and a server has a service curve *β*(*t*) if $R^{*}(t)\geq (R⊗\beta )(t)$, where *R*^*^ is the output flow for *R*. In this case, *α*^*^(*t*) = (*α*∅*β*)(*t*) is the arrival curve for *R*^*^ and can be used for the following aggregate behaviors.

The worst case latency for flow *R* when it is served by an output port with a service curve *β*(*t*) is the maximum horizontal difference between *α*(*t*) and *β*(*t*) and can be formally defined by:

$$h(\alpha ,\beta )=\underset{s\geq 0}{\sup}\left(\inf\left\{\tau \geq 0|\alpha (s)\leq \beta (s+\tau )\right\}\right).$$

The end-to-end worst-case delay for flow *R* is the sum of all latency bounds in the corresponding output ports along its transmission paths.

If a flow strictly obeys the leaky bucket model [39], such as a VL in the AFDX network, its arrival curve can be simplified as:

$$\alpha_{\sigma ,\rho }(t)=\sigma +\rho \times t(t\geq 0)$$

where *σ* is the maximal burst and *ρ* is the long-term bit rate. When the bandwidth allocation gap *BAG* and the maximal frame length *S_max_* of VL are considered, these two parameters can be specified as *σ* = *S_max_* and *ρ* = *S_max_* /*BAG*. A typical example for the service curve is the rate-latency service curve with *C* as the service rate (such as 100Mbps) and *T* as the maximal technology latency of the switch (such as 10 μs). Thus, it can be defined as:

$$\beta_{R,T}(t)=C{\left[t-T\right]}_{0}^{+}$$

According to these models, the worst-case latency *D* can be easily calculated out and the uncertainty of flow’s arrival for the next aggregate nodes can be represented by a change of burstiness, thus the arrival curve for *R*^*^ should be restricted by [39]:

$$\alpha_{\sigma ,\rho }^{*}(t)=\sigma +\rho \times D+\rho \times t(t\geq 0)$$
